# miR-484: A Potential Biomarker in Health and Disease

**DOI:** 10.3389/fonc.2022.830420

**Published:** 2022-03-09

**Authors:** Yin-zhao Jia, Jing Liu, Geng-qiao Wang, Zi-fang Song

**Affiliations:** ^1^ Department of Hepatobiliary Surgery, Union Hospital, Tongji Medical College, Huazhong University of Science and Technology, Wuhan, China; ^2^ Key Laboratory of Coal Science and Technology of Ministry of Education, College of Chemistry and Chemical Engineering, Taiyuan University of Technology, Taiyuan, China

**Keywords:** cancer, physiological conditions, metastasis, proliferation, apoptosis

## Abstract

Disorders of miR-484 expression are observed in cancer, different diseases or pathological states. There is accumulating evidence that miR-484 plays an essential role in the development as well as the regression of different diseases, and miR-484 has been reported as a key regulator of common cancer and non-cancer diseases. The miR-484 targets that have effects on inflammation, apoptosis and mitochondrial function include SMAD7, Fis1, YAP1 and BCL2L13. For cancer, identified targets include VEGFB, VEGFR2, MAP2, MMP14, HNF1A, TUSC5 and KLF12. The effects of miR-484 on these targets have been documented separately. Moreover, miR-484 is typically described as an oncosuppressor, but this claim is simplistic and one-sided. This review will combine relevant basic and clinical studies to find that miR-484 promotes tumorigenesis and metastasis in liver, prostate and lung tissues. It will provide a basis for the possible mechanisms of miR-484 in early tumor diagnosis, prognosis determination, disease assessment, and as a potential therapeutic target for tumors.

## Introduction

## MicroRNAs Biological Origin and Features

According to current knowledge, about 90% of the human genome is transcribed, and less than 3% of the genome is capable of encoding proteins, yet most of the genome produces non-coding RNA (ncRNA) (1). MicroRNA (miRNA), with a length of about 22 nucleotides(nt), is a member of the regulatory and small ncRNAs. miRNAs are widely observed in eukaryotes and remain highly conserved and homologous, suggesting that miRNAs have the same regulatory mechanisms during development in different organisms (2, 3). In addition, miRNA expression maintains a dynamic regulatory state at different developmental time points or in diverse cells and tissues (4–6). Although only about a thousand miRNAs have been identified, they regulate close to 30% of gene expression, which is closely related to the phenomenon of miRNA motif clustering. miRNAs bind to target RNAs through complementary pairing at the 5’ end (known as seed sequences) and down-regulate gene expression at the post-transcriptional level consisting mainly of mRNA degradation or translational repression (7). In recent years, some scholars have proposed that miRNAs can activate gene transcriptional expression by binding to enhancers and changing the chromatin state of enhancers, and named these miRNAs as Nuclear Activating miRNA (NamiRNA) and proposed a new regulatory model of “NamiRNA-enhancer-gene activation” (8–10). In conclusion, miRNAs have a dual function of repressing gene expression in the cytoplasm and activating gene transcription in the nucleus.

Since miRNAs regulate multiple biological functions in many different specific pathways or physiological processes, abnormal miRNA expression has great potential in disease diagnosis and prognosis. The role of miRNAs in cancer is divided into oncogenes, tumor suppressor genes, and mixed effects. For example, miR-126 contributes to the development of chronic granulocytic leukemia by promoting the downregulation of B-cell differentiation genes and regulating the ability of myeloid cells to adhere and migrate (11). However, deletion or downregulation of miR-15 and miR-16 gene fragments are present in more than half of the B-cell chronic lymphocytic leukemia (12). The miR-29 family plays an oncogenic role in colorectal cancer (13) nevertheless high expression of miR-29 advances acute myeloid leukemia disease progression (14). miRNAs are also involved in the regulation of embryonic development. The first identified miRNAs, miRNA-lin4 and let-7, are involved in controlling the timing of nematode development. In addition, miRNAs are also engaged in the regulation of immune response, glucolipid metabolism and ischemia-hypoxia related pathways, such as ankylosing spondylitis (15), diabetes (16), non-alcoholic fatty liver diseases (17) and myocardial infarction (18).

In summary, individual miRNA could regulate complex physiological or disease phenotypes by modulating entire functional networks. Screening specific miRNA molecules as biomarkers for assessing disease progression and prognosis, especially targeting miRNA therapy is not only challenging but also promising for some diseases.

## Important Characteristics of miR-484

**Figure 1:**
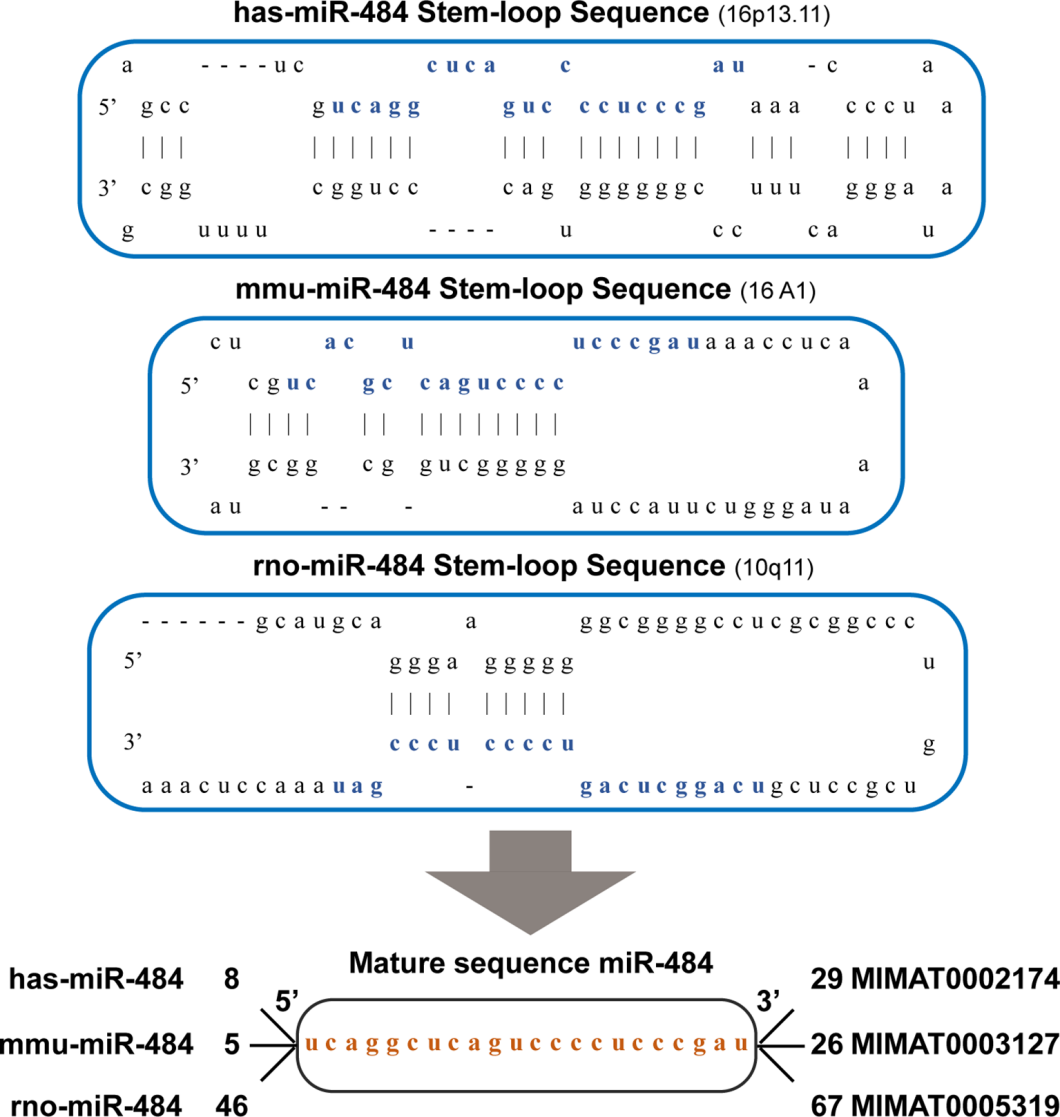
The sequence structure of the miR-484. Hsa-miR-484, mmu-miR-484 and rno-miR-484 are located on chromosome 16 (chr16: 15643294-15643372), chromosome 16 (chr16: 14159626-14159692) and chromosome 10(chr10: 27845-27921and 908408-908484). They all have the same and only one miR-484 mature sequence.

**Figure 2:**
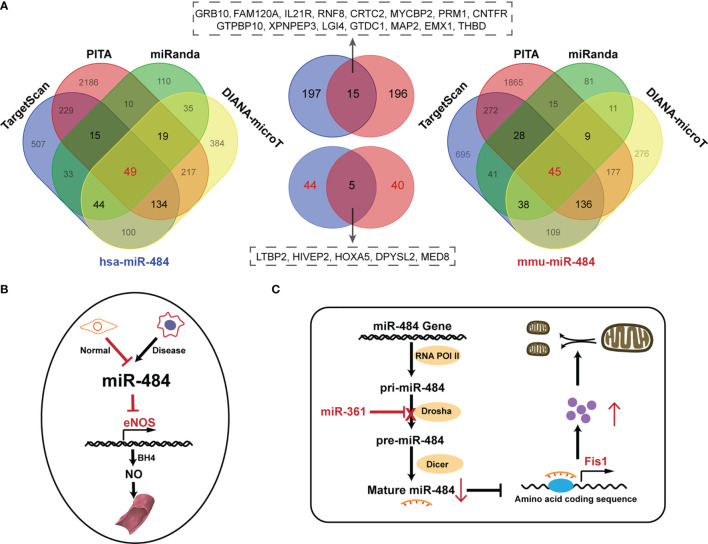
Prediction of miR-484 downstream targets and the role of miR-484 in physiological states. **(A)** The Wayne diagram shows the results of hsa-miR-484 and mus-miR-484 target prediction by four miRNA-related databases. In addition, a cross-set of common downstream targets for mouse and human. Left: hsa-miR-484 predicted target results. Right: mus-miR-484 predicted targets. Middle top: cross-set of three databases common to human (blue) and mouse (red) targets. Middle bottom: cross-set of four databases common to human (blue) and mouse (red) targets. **(B)** Mockup shows the mechanism of miR-484 involvement in maintaining the function of Ecs. **(C)** The mechanism of miR-484 involvement in mitochondrial function division in cardiomyocytes.

miR-484 is not highly conserved in mice, rats, and humans and located in the Meiosis arrest female 1 (MARF1) promoter region on host gene chromosome 16p13.11 in human and mouse and on rat chromosome 10q11, respectively. Notably, the human chromosome 16p13.11 microduplication may be pathogenic for the nervous system (19, 20). In this review, we focus on the essential functions of miR-484 in health and various diseases. In human, mice and rat, mature miR-484 is composed of 22 nucleotides **(** **Figure 1** **)**. However, miR-484 stem-loop sequences are not identical among various genera **(** **Figure 1** **)**. Our initial screening of miR-484 downstream targets by classical miRNA target gene prediction tools (TargetScan, PITA, DIANA-micro and miRanda) revealed 45 and 49 target genes for miR-484 in mouse and human species, respectively **(** **Figure 2A** and **Supplementary Tables 1–2** **)**. Further analysis revealed that only 5 of the above target genes were targeted by miR-484 in both human and mouse species **(** **Figure 2A** **)**. In addition, as shown in **Table 1** is a comprehensive list of miR-484 downstream targets that have been validated to date. The physiological functions of miR-484 in mammals are multifaceted and involve endoplasmic reticulum stress, oxidative stress, inflammation, cell proliferation and apoptosis. Besides, several reports have confirmed the abnormal expression of miR-484 in both tissue and serum specimens from clinical patients (29, 30, 32). The modalities and regulatory mechanisms of miR-484 regulation of downstream target genes in different types of cells and their microenvironments are unique. First, mutations in the bta-miR-484 seed sequence neo-SNPs (G4693T) directly lead to increased expression of heat shock transcription factor 1 (HSF1) while affecting the heat stress resistance response in cows (21, 22). Secondly, miR-484 has a unique type of regulation in the cytoplasm. miR-484 reduces Fis1 protein expression levels by binding to the amino acid coding sequence of Fis1 and inhibiting its translation (48). Next, unlike the former, miR-484 undergoes its own maturation disorder in the nucleus.miR-361 can bind to the primary transcript of miR-484 (pri-miR-484) resulting in the inability of Drosha to process into pre-miR-484 in the nucleus (23). Finally, miR-484 regulates its own expression in the nucleus through epigenetic mechanisms. Hu et al. demonstrated that the reduction of DNA methyltransferase DNMT1 recruited by EZH2 in cervical cancer cells resulted in decreased CpG methylation of the miR-484 promoter raising the expression level of miR-484 (32). Consistency with the former, hypermethylation at the CpG island site of the miR-484 promoter in microsatellite instability colorectal cancer significantly downregulates miR-484 production (33). In apart from the above mechanisms, mature miRNAs compete with long-stranded non-coding RNAs (lncRNAs) to bind the 3’-UTR of target mRNAs and indirectly inhibit the negative regulation of target genes by miRNAs, such as lncRNA Ttc3-209 in human retinal cells (24), PGM5-AS1 in colorectal cancer (28), TMEM220-AS1 in hepatocellular carcinoma cells (29), THAP9-AS1 in pancreatic ductal adenocarcinoma (30) and PCED1B-AS1 in Clear Cell Renal Cell Carcinoma (27), which are considered as ceRNAs, by competitively inhibiting miR-484 binding and upregulating the translation of target proteins.

**Table 1:** Validated targets of the miR-484.

	miRNAs	Downstream Target Gene	Upstream Target Gene	Feature	Effect	Tissue or cell line	PMID
1	bta-miR-484	HSF1		NA-binding transcription factor	Transcriptional activation of the heat shock response	Chinese Holstein cattle	(21, 22)
2	pri-miR-484		miR-361 MDRL		Inhibition of mature miR-484 production	Neonatal mouse cardiomyocytes	(23)
3	mmu-miR-484 hsa-miR-484	Fis-1	Foxo3a	Mitochondrial fission Transcriptional activator which triggers apoptosis	Apoptosis	Mouse cardiomyocytes Human adrenocortical cancer cells (H295R cell)	(23)
4	mmu-miR-484	Wnt8a	lncRNA Ttc3-209	Transmembrane receptor	Apoptosis	Retinal ganglion cells	(24)
5	mmu-miR-484			LncRNA: ceRNA	Apoptosis	Retinal ganglion cells	(24)
6	mmu-miR-484	BCL2L13		Apoptosis facilitator	Apoptosis	Mouse brain tissue	(25)
7	rno-miR-484	SMAD7		TGF-β receptor antagonist	Apoptosis	Rat heart tissue	(26)
8	hsa-miR-484	ZEB1	lncRNA PCED1B-AS1	Transcriptional repressor	Proliferation, migration and EMT	Human clear cell renal cell carcinoma	(27)
9	hsa-miR-484		lncRNA PGM5-AS1		Proliferation, migration and EMT	Human colorectal cancer (SW480 and HCT116 cells)	(28)
10	hsa-miR-484	MAGI1	lncRNA TMEM220-AS1	Membrane associated guanylate kinases	Proliferation, migration and EMT	Human liver tumor tissue	(29)
11	hsa-miR-484	YAP	lncRNA THAP9-AS1	The critical downstream regulatory target in the Hippo signaling pathway	Promotes THAP9-AS1 transcription to form a feed-forward circuit	Human pancreatic ductal adenocarcinoma	(30)
12	hsa-miR-484	eNOS		Nitric oxide synthase	Evokes endothelial dysfunction	Human umbilical endothelial cells (HUVECs)	(31)
13	hsa-miR-484	MMP14 HNF1A		Endopeptidase Transcriptional activator	Regulates the WNT/MAPK and TNF signaling pathway	Human cervical cancer tissue	(32)
14	hsa-miR-484		EZH2 DNMT1	Transcriptional repression Methylates hemimethylated DNA.	CpG methylation of miR-484 promoter	Human cervical cancer tissue	(32)
15	hsa-miR-484	CD137L		Tumor necrosis factor family	Attenuated IL-8 production	Human colorectal cancer	(33)
16	rno-miR-484	YAP1		Transcriptional coactivator	Apoptosis	Rat cardiomyocytes (H9c2)	(34)
17	rno-miR-484		LINC00339	LncRNA	Inhibit proliferation Promotes apoptosis	Rat cardiomyocytes (H9c2)	(34)
18	Mmu-miR-484	PCDH19		Potential calcium-dependent cell-adhesion protein	promotes neurogenesis	Mouse cortical progenitor Mouse cortical neuron	(20)
19	Hsa-miR-484	CCL-18		CC-type chemokine	Suppress cell proliferation	Human GC tissue	(35)
0	Hsa-miR-484		ZFAS1	LncRNA	Suppress cell proliferation and invasion	Human CRC tissue Human CRC cell lines	(36)
21	Hsa-miR-484	KLF12	LINC00239	Transcriptional repression	Suppress cell proliferation and invasion	Human CRC tissue Human colon epithelium cell line (FHC) Human CRC cell lines	(37)
22	Hsa-miR-484		CircADAMTS13	circRNA	Promotes tumorigenesis	Human tumor and matched peritumor tissues	(38)
23	Hsa-miR-484	TUSC5		Tumor suppressor	Promotes tumorigenesis	Paired HCC and adjacent normal tissue	(39)
24	Hsa/mus-miR-484	SAMD9		Endosome fusion facilitator,	Promotes tumorigenesis	Human HGDN specimen Mouse liver tissue	(40)
25	Hsa-miR-484	YAP	THAP9-AS1	Transcriptional coactivator LncRNA	Tumour-suppressive	Female Balb/C athymic nude mice	(30)
26	Hsa-miR-484	PSMG1		Promotes proteasome assembly	Promotes recurrence and migration	Human prostate cell lines	(41)
27	Hsa-miR-484	Apaf-1		Apoptotic protease-activating factor	Promotes tumorigenesis	Human NSCLC tissues Human NSCLC cell lines	(42)
28	Hsa-miR-484	OLA1		Hydrolyzes ATP and GTP	Potential biomarkers	Human nasopharyngeal cell lines	(43)
29	Hsa-miR-484	KLF4		Kruppel family of transcription factors	Reduce tamoxifen resistance	The human breast carcinoma cell lines	(44)
30	Hsa-miR-484	CDA		Key regulator of deoxyuridine conversion	Improves chemosensitization and cell proliferation	The human breast carcinoma cell lines	(12)
31	Hsa-miR-484	ZEB1 SMAD2		Promotes cell growth, migration, invasion, and EMT	Suppression of the malignant behavior	Human cervical cancer tissue specimens and cell lines	(45)
32	Hsa-miR-484	VEGFB VEGFR2		Involved in angiogenesis	Improves chemotherapy sensitivity	Human ovary carcinoma tissues	(46)
33	Hsa-miR-484	MAP2	c-Myc	A-kinase anchoring proteins	Augment the tumor-initiating capability	Human glioma tissues and cell lines	(47)

Hypoxia, chronic inflammation, immunosuppression, ionizing radiation and other cellular microenvironmental factors can affect miRNA expression. For example, the pathological process of ischemic perfusion injury induces miR-484 expression in retinal ganglion cells (24), cardiac muscle (26, 49, 50) and brain tissue (25). Low-dose ionizing radiation exposure induces downregulation of miR-484 expression in small cell lung cancer cells (51, 52). Chronic inflammation stimulates keratin-forming cells and endometrial cells to upregulate miR-484 (53, 54).

## Physiological Roles of miR-484

### miR-484 Mediates Endothelial Cell Vulnerability

Normally, endothelial nitric oxide synthase (eNOS) is activated by binding to tetrahydrobiopterin (BH4) in endothelial cells (ECs) and catalyzes the synthesis of nitric oxide (NO) from arginine and oxygen, leading to vasodilation. However, reactive oxygen species (ROS) degradation of BH4 leads to eNOS inactivation causing EC dysfunction. miR-484 and miR-93 in ECs are able to target the seed sequence of the 3’UTR of eNOS and Krüppel-like factor 2 (KLF2) mRNAs and repress protein transcription (31, 55). The sustained low expression of miR-484 in ECs under physiological conditions protects against endothelial injury by pulsatile shear (PS) and oscillatory shear (OS). Interestingly, there was a high enrichment of miR-484 in diseased endothelial progenitor cells (56). Furthermore, miR-484 is a molecular marker of carotid plaque development and rupture vulnerability (56). It is reasonable to speculate that miR-484 plays an important role in maintaining vascular endothelial cell homeostasis and inhibiting endothelial dysfunction **(** **Figure 2B** **)**.

### miR-484 Affects Mitochondrial Biological Function and Morphology

The physiological balance of mitochondria to maintain the fused and divided state provides important energy for cellular life activities. miR-484 was ability to alleviate the reduced I/R mitochondrial membrane potential in cardiomyocytes by decreasing the level of apoptosis (26). In addition, a long-stranded noncoding RNA called mitochondrial dynamics-related lncRNA (MDRL) regulates mitochondrial division and apoptosis by targeting miR-361 to block pri-miR-484 binding to Drosha, leading to impaired production of mature miR-484 (23). Recently, it has been demonstrated that the Foxo3a-miR-484-Fis1 signaling axis directly regulates the mitochondrial division program in cardiac myocytes (48). Based on the effect of miR-484 in regulating mitochondrial morphology and function, it is expected to be an important research target for mitochondria-related diseases in the future. **(** **Figure 2C** **)**.

### miR-484 Regulates Glucolipid Metabolism

miRNAs play an active role in glucose synthesis and lipid metabolism. Elevated glucose significantly downregulated miR-484 levels in pancreatic β-cells, suggesting that miR-484 may be a regulator of insulin gene expression (57). Raitoharju et al. revealed (58) that hsa-miR-484 is strongly associated with insulin resistance-related metabolites. RNA-seq analysis of serum circulating miRNA (c-miRNA) revealed that miR-484 was significantly up-regulated in obese children of born small for gestational age (SGA) and appropriate for gestational age (AGA) (59). Surprisingly, a study confirmed that circulating miR-484 and miR-378 were most significantly negatively associated with BMI and visceral fat content, and even GO analysis showed that miR-484 and miR-378 target genes are closely associated with carbohydrate and lipid metabolism (60). Further, Miyamoto et al. constructed palmitate-induced endoplasmic reticulum stress conditions in hepatocytes *in vitro* and found that significant downregulation of miR-484 is closely associated with lipoapoptosis (61). In summary, miR-484 may be more promising and has potential for study in insulin signaling, glucose transport, insulin resistance and lipid metabolism.

### miR-484 Contributes to Neurological Function

Some specific miRNAs in the central nervous system (CNS) are of vital concern in various aspects involved in central development, neuronal differentiation and synaptic shaping (62–64). Luceri et al. identify that mmu-miR-484 upregulation in the cortex and cerebellum regions may be associated with changes in cognitive, motor and emotional behavior in mice (65). Interestingly, mmu-miR-484 is involved in stress resilience through the regulation of Map2k4 and Pik3r1in prefrontal cortical regions (66). In other, it has been documented that the predicted targets of miR-484 are enriched in protein co-expression modules of synaptic transmission and long-term synaptic plasticity regulation, which may be relevant to cognitive function (67). Consistent with the former results, miR-484 target genes were mainly concentrated in cognitive function-related genes and neurotrophin signaling genes were significantly enriched (68). It is reasonable to speculate that miR-484 has a potential regulatory role in neurological cognitive function.

## The Role of miR-484 in Non-Cancerous Diseases

### Cardiovascular Diseases

#### Acute Myocardial Infarction

Acute myocardial infarction (AMI) has constituted one of the major hazards to the public, featuring high incidence, mortality and disability. The time from the onset of infarct symptoms to the onset of fibrous reperfusion is a key determinant of successful recanalization and survival. Nevertheless, there is no effective treatment for the reperfusion process that naturally induces cardiomyocyte death. Some cardiac-specific miRNAs have gained attention as potential therapeutic targets due to their role in gene regulation during disease progression. For example, MRG-110, which targets miR-92a, entered clinical trials because it significantly stimulated revascularization and healing and alleviated ischemic injury in the heart (69). miR-484 is most abundantly expressed in the heart and dysregulated in human ischemic heart samples, suggesting a potential close relationship between miR-484 and cardiac ischemia-related diseases (70).TGF-β-SMADs signaling axis as one of the major pathways of myocardial ischemia-reperfusion injury (MI/R) (71, 72). Notably, Liu et al. revealed that rat-miR-484 directly targets SMAD7 to inhibit apoptosis to protect against MI/R in rats (26). Furthermore, whether the relationship of miR-484 indirectly regulating SMAD2 in cervical cancer tissues (45) also exists with cardiac tissues or even involved in MI/R remains to be further tested. Particularly, miR-484, which was shown to be a Foxo3a trans-activator, exerts a protective importance by directly targeting Fis1 during MI/R (48) **(Table 2)**. Moreover, ability to tolerate high-intensity exercise in patients recovering from AMI has a significant positive correlation with circulating miR-484 levels (49). Combining these results revealed that both MI/R process tissue and circulating miR-484 act as protective factors to slow down impaired cardiomyocyte function and accelerate the recovery process, promising to already be a powerful diagnostic or prognostic biomarker and a particularly promising drug target candidate for therapeutic applications in cardiovascular disease.

**Table 2:** The role of miR-484 in Non-Cancerous Diseases.

	Disease	Species and tissue or cell type	Alteration of miR-484 expression	Method for miR-484detection	Target gene	Method for target validation	Biological function	Sample size in clinical studies	Ref
1	MI/R	Rat heart tissue	down-regulation	qRT-PCR	SMAD7	qRT-PCR and the luciferase reporter gene assay	Anti-inflammatory Anti-apoptotic Protection of mitochondria	N/A	(26)
2	MI/R	Mouse heart tissue	down-regulation	qRT–PCR	Fis-1	qRT-PCR, WB and Luciferase assays	Inhibit mitochondria fission	N/A	(48)
3	MI/R	Human BS (Japan)	up-regulation	qRT–PCR	N/A	N/A	High exercise capacity after MI/R	AMI patients(n=20) and healthy control subjects (n=5)	(49)
4	AS	Human postbifurcation carotid plaques (Pennsylvania)	up-regulation	Affymetrix GeneChip microRNA Array and qRT–PCR	DACH1	N/A	Associated with plaque fragility after carotid bifurcation	symptomatic (n=9) and asymptomatic patients (n=9) with carotid stenosis	(73)
5	AS	Human BS (American)	up-regulation	qRT–PCR	N/A	N/A	Induction of endothelial dysfunction	patients with CAD (n = 56) and HCs (n = 10)	(31)
6	AS	Human plasma and EPCs of patients with CAD	up-regulation	smRNA-seq qRT–PCR	N/A	N/A	Cannot inhibit VEGF expression and EPC activity	N/A	(56)
7	SIC	Rat cardiomyocytes (H9c2)	up-regulation	qRT–PCR	YAP1	qRT-PCR, WB and the luciferase reporter gene assay	Promoted cell viability Decreased apoptosis and inflammation	N/A	(34)
8	DIC	Rat cardiomyocytes (H9c2)	down-regulation	qRT–PCR	LINC00339	the luciferase reporter gene assay	Promot proliferation Inhibit apoptosis	N/A	(74)
9	16p13.11 microduplication syndrome	Mouse cortical progenitor Mouse cortical neuron	up-regulation	qRT–PCR	PCDH19	the luciferase reporter gene assay	promotes neurogenesis	N/A	(20)
10	Cerebral injury-related diseases	Mouse brain tissue	down-regulation	qRT–PCR	BCL2L13	the luciferase reporter gene assay	Inhibit apoptosis	N/A	(25)
11	Psoriasis	Human epidermal keratinocyte (China)	down-regulation	LncRNA and mRNA microarray and construction of the competing endogenous RNA (ceRNA) network	MLH3, lncSNX10-2:8, lnc-MNX1-5:1, lnc-1QCD-1:1	N/A	N/A	15 age-matched and gender-matched (n=15) healthy skin control 15 patients with progressive psoriasis vulgaris (n=15)	(53)
			up-regulation		DCTN3 Lnc-AGXT2L1-2:2				
12	Hepatitis B virus	Human dendritic cells (India)	up-regulation	qRT–PCR Unsupervised hierarchical clustering and principal component analyses	N/A	N/A	N/A	HBV infection and liver disease namely, immune active (IA; n = 20); low replicative (LR; n = 20); HBeAg negative (n = 20); acute viral hepatitis (AVH, n = 20) and healthy controls (n = 20).	(75)
13	Hepatitis C virus	Human BS (Egyptian)	up-regulation	qRT–PCR	N/A	N/A	N/A	Egyptian patients with HCV liver fibrosis(n=47), HCV-cirrhosis(n=40), HCV- HCC(n=41) Healthy controls(n=40)	(76)
14	Dengue	Vero cells	down-regulation	qRT–PCR	DENV RNA	qRT–PCR The RNAhybrid program MicroInspector	virus replication	Vero cells (CCL-81) Mosquito C6/36 HT cells	(77)
15	Tuberculosis	Human serum-derived exosomes (Iran)	up-regulation	qRT–PCR	N/A	N/A	N/A	Patients with TB(n=25) Healthy controls with a negative history of TB (n=25)	(78)
16	Leprosy	Human leprosy skin lesions (Brazil)	up-regulation	qRT–PCR	FASN	The genes selected from the miRNA/mRNA analysis were submitted to pathway enrichment analysis by using the ReactomeFIViz plugin from the Cytoscape software	N/A	Leprosy lesions (TT = 10, BT = 10, BB = 10, BL = 10, LL = 4, R1 = 14, and R2 = 10) Healthy control (n = 9)	(79)

BS, Blood sample; CAD, coronary artery disease; EPC, endothelial progenitor cell; IA, immune active; LR, low replicative; AVH, acute viral hepatitis; TT, tuberculoid; BT, borderline tuberculoid; BB, borderline borderline; BL, borderline lepromatous; LL, lepromatous; R1, type 1 reaction; R2, type 2 reaction.

NA, Not answered.

#### Atherosclerosis

Atherosclerosis (AS) is one of the most common diseases of the cardiovascular system. Its pathogenesis is complex and is mainly characterized by plaques formed by lipid accumulation, fibrous tissue proliferation and calcium deposition in the intima.

High-risk factors such as inflammation, blood flow shear and hypertension accelerate impaired endothelial cell (EC) function as one of the main pathological processes in AS. miRNA-induced EC dysfunction and high expression of adhesion molecules accelerate AS plaque formation (80). For example, miR-34a and miR-216a inhibited silent information regulator 1 (SIRT1) (81) and SMAD3 (82) expression levels promoting ECs senescence and adhesion, respectively. Caparosa et al. collected identical post-bifurcation rupture-prone plaques from symptomatic and asymptomatic patients with carotid stenosis by microarray assay of miRNA and mRNA expression profiles to finally screen for differentially expressed miRNAs. surprisingly, miR-484 was included, and DACH1 screened as a high confidence mRNA target for miR-484 (73). Given the instability of post-bifurcation plaques, miR-484 is expected to be a molecular marker of carotid plaque rupture vulnerability. Furthermore, the role of DACH1 on vascular endothelial cell development and migration is supported by evidence (83, 84). In another study, it was demonstrated that high expression of DACH1 in mouse cardiac endothelial cells (ECs) significantly promoted coronary artery differentiation and significantly improved survival after myocardial infarction in mice (85). The potential of miR-484 to target DACH1 molecules to regulate arterial EC cell activity and differentiation, among others, remains to be further tested. Moreover, abnormal levels of exosomal miRNA in serum of AS patients have been demonstrated (86). Notably, Wang et al. found by small RNA sequencing (smRNA-seq) that miR-484 showed high enrichment in both diseased endothelial progenitor cells and plasma from patients with coronary atherosclerotic heart disease (56) **(** **Table 2** **)**. The literature has reported that miR-484 is elevated in the serum of CAV patients compared to EC healthy individuals, which is consistent with the former results (31). Unfortunately, miR-484 cannot inhibit the expression of VEGF, significantly involved in the progression of AS pathogenesis, and modulate the cell activity of ECs (56). In summary, miR-484 increases the fragility of vascular plaques, but its specific mechanism for regulating endothelial cell damage is still unclear.

#### Sepsis Induced Cardiomyopathy

Sepsis Induced Cardiomyopathy (SIC) is clinically defined as sepsis in combination with cardiac dysfunction including myocardial depression and acute heart failure. Current studies have shown that the pathogenesis of SIC involves a multifactorial role, mainly including the release of large amounts of inflammatory factors, imbalance of calcium homeostasis, mitochondrial dysfunction (87), apoptosis and cell death, and a complex association between these factors (88). In addition, miRNAs have been shown to play a potential role in the pathophysiology and clinical diagnosis and even prognosis of SCI. For example, miR-539-5p targets IRAK3 to inhibit inflammatory release to alleviate LPS-induced sepsis (89). miR-223 knockout mice significantly exacerbated SIC-induced cardiac dysfunction (90). Interestingly, LPS treatment of H9C2 cells promoted miR-484 to negatively regulate YAP1 expression. Interestingly, LPS treatment of H9C2 cells promoted miR-484 to negatively regulate YAP1 expression (34) **(** **Table 2** **)**. miR-484 inhibitor significantly improved LPS-induced cell viability and apoptosis, whereas YAP1 knockdown reversed the effect of miR-484 inhibitor (34). YAP1, a key downstream regulatory target in the Hippo pathway, is closely associated with immune disorders and inflammatory diseases (91). Endothelial YAP1 deficiency increases cardiovascular dysfunction in a microbial sepsis model (92).

#### Drug-Induced Cardiomyopathy

Cardiotoxicity caused by antineoplastic drugs is statistically the second leading cause of death in long-term oncology survivors (93). Anthracyclines have been reported to be the most common drugs causing cardiotoxicity (94). It has been proposed that doxorubicin, one of the anthracycline antitumor drugs, significantly upregulates LINC00339 after treatment of cardiomyocytes (74) **(** **Table 2** **)**. Further studies revealed that LINC00339 directly inhibits miR-484 expression promoting cardiotoxic injury (74). The present results provide new biomarkers and therapeutic targets for dox-induced cardiotoxicity.

### Neurological Diseases

#### 16p13.11 Microduplication Syndrome

16p13.11 microduplication syndrome is an autosomal dominant disorder caused primarily by a mesenchymal duplication of 16p13.11 with a duplication region containing two genes associated with the neurobehavioral phenotype nucleus distribution gene homolog 1 (NDE1) and asparaginase1 (NTAN1), respectively **(** **Table 2** **)**. The disorder manifests itself primarily as a syndrome associated with behavioral abnormalities, developmental delays, congenital heart defects and skeletal abnormalities, and other clinical features of the syndrome. Surprisingly, human and mouse miR-484 are located exactly in the mutated gene sequence, which in part suggests a link between miR-484 in neurodevelopment. In addition, it has been documented that NDE1 is a potential downstream target of miR-484 (95). Fujitani (20) and Khattabi (19) proposed that the 16p13.11 microduplication is strongly associated with miR-484. The mechanism of genetic variation therein confirms that imbalance in the expression of mature mmu-miR-484 and protocadherin-19 (PCDH19) affects neurogenesis (19) **(** **Table 2** **)**. PCDH19 mutant mice exhibit significant synaptic dysfunction and cognitive impairment. In summary, we believe that miR-484 has important potential research value in neurological development.

#### Alzheimer’s Disease

Alzheimer’s disease (AD) is a heterogeneous central neurodegenerative disorder, and its pathogenesis is mainly related to the abnormal deposition of amyloid Aβ and Tau protein hyperphosphorylation causing impaired neuronal function and synaptic transmission. Aberrant miRNA expression in the CNS affects regulatory target genes causing CNS dysfunction. Cai et al. first constructed miRNA-seq on Alzheimer’s disease model mice (APPswe/PS1ΔE9 transgenic mice) and found aberrant mmu-miR-484 expression (68). Analysis of the abnormal upregulation of miR-484 in bipolar disorder exosomes using the Kyoto Gene and Genome Encyclopedia (KEGG) pathway with DIANA-miRPath v3.0 revealed that it was mainly associated with the PI3K/Akt signaling pathway (96) **(** **Table 2** **)**. Interestingly, the PI3K-Akt signaling pathway may be an important target for AD therapy (97). In addition, it has been proposed that low levels of miR-484 are associated with rapid cognitive decline (67). Further studies confirmed that the downstream predicted target functions of miR-484 are mostly associated with motor and cognitive behavior (65) and neurotransmitter synaptic transmission (68, 98). Up to now there is a paucity of literature related to miR-484 and the pathogenesis of AD, but it is undeniable that miR-484 is closely related to the occurrence of AD-related cognitive functions.

#### Cerebral Injury-Related Diseases

The current effective methods for ischemic cerebral infarction include intravenous thrombolysis and mechanical embolization. Nevertheless, reperfusion injury is the main cause of poor healing and high morbidity and mortality in ischemic stroke. Although the mechanism of injury and its complexity but the role of neuronal apoptosis-induced cerebral ischemia/reperfusion injury cannot be ignored (99).

A recent study initially demonstrated that cerebral ischemia/reperfusion injury in mice induced a significant downregulation of mmu-miR-484 expression (25). Further studies revealed that miR-484 acts as a neuroprotective factor and inhibits BCL2L13 overexpression to attenuate neuronal apoptosis (25) **(** **Table 2** **)**. Similarly, miR-484 was reported to exert a protective effect by inhibiting Wnt8a mRNA translation to attenuate apoptosis during retinal ischemia re-injury (24). In addition, miR-484 levels in blood are valuable for the diagnosis of the degree of traumatic brain injury (100).

### Skin Diseases

miRNAs play an integral role in skin and appendage genesis, and skin morphogenesis is regulated by the discontinuous differential expression of miRNAs. Its role in coordinating the proliferation and differentiation of the epidermis is attracting increasing attention from domestic and international dermatologists. Three miRNAs (miR- 203, miR-146a and miR-125b) currently associated with psoriasis are involved in the natural immune response and the TNF-α pathway (101). Wang et al. used microarrays to compare LNCRNA and mRNA expression in keratin-forming cells from patients with psoriasis and healthy patients, showing that the miRNA maximally linked to LNCRNA and mRNA was miR-484, adding some theoretical basis for miR-484 in the mechanism of psoriasis (53) **(** **Table 2** **).** Notably, the ability of miR-484 to negatively regulate the TNF signaling pathway in cervical carcinogenesis development was previously reported (32).

### Infection-Related Diseases

#### Viral Infections

Hepatitis B virus (HBV) infection is one of the global health problems. The interaction of host genes as well as the respective miRNAs encoded by viral genes affects the replication and transcription of HBV. Differentially expressed miRNAs may be associated with persistent HBV infection. Singh et al. revealed (75) that miR-484 is significantly upregulated in dendritic cells of patients with acute viral hepatitis B compared to healthy individuals and is accompanied by alterations in this antigen processing and delivery related target genes. Besides, hepatitis C virus infection (HCV)-mediated liver fibrosis and cirrhosis are also receiving increasing attention from clinicians. El-Maraghy et al. (76) examined miR-484 expression levels in plasma from patients with HCV infection-induced liver fibrosis, cirrhosis, hepatocellular carcinoma (HCC) and healthy volunteers, and confirmed that miR-484 showed significantly low expression in the advanced fibrosis stage, however, it was significantly upregulated in the mild fibrosis stage and in liver cirrhotic and HCC **(** **Table 2** **)**. From another perspective, miR-484 could be used for staging prognosis and early diagnosis of HCV-induced progression of liver lesions. Similarly, circulating miR-484 in plasma may serve as a useful biomarker for predicting Ebola virus vaccine VSV-EBOV-induced induction of immunogenicity (102). On the other hand, miR-484 and miR-744 were able to bind to a conserved region in the 3’ -untranslated region (3’-UTR) of dengue virus RNA (DENV-RNA) to exert antiviral effects by inhibiting viral gene replication (77).

#### Mycobacterium Bifidum Infection

Tuberculosis (TB) is a chronic infectious disease caused by infection with Mycobacterium tuberculosis. Globally, TB is the leading cause of death from a single source of infection (surpassing AIDS). Patients with active tuberculosis (ATB) are a major source of TB infection, making rapid and accurate diagnosis of ATB particularly important to control disease transmission and improve treatment outcomes. Several studies have shown that miRNAs are associated with MTB activity in the host. Alipoor et al. demonstrated that miR-484, miR-425, and miR-96 expression was elevated in serum exosomes of patients with TB compared to healthy subjects, and miR-484 was particularly elevated (103). Similarly, miR-484 expression was found to be upregulated by infection with Bacillus Calmette-Guérin (BCG) human macrophages (78) **(** **Table 2** **)**. Interestingly, the expression level of miR-484 showed a significant positive correlation with the degree of smear positivity. To further test the correlation between specific miRNAs and the active phase of tuberculosis, the alteration of miR-484 and miR-425 combination had considerable predictive value for tuberculosis by ROC curve analysis (103) In addition, as a representative of nontuberculous mycobacteria, leprosy caused by Mycobacterium leprae infection shows clinically different histopathological changes caused by differences in the organism’s response to immunity. Leprosy is classified according to the intensity of cellular immunity into type I reactions (R1) which are Mycobacterium leprae mediated immune reactions or delayed metaplasia, and type II reactions (R2) which occur in antigen, antibody complex metaplasia, i.e. vascular inflammatory reactions. Cleverson T Soares et al. found that hsa-miR-484 was significantly more expressed in skin lesion tissues of R2 patients than healthy individuals and predicted a potential downstream target molecule as FASN (79).

## The Role of miR-484 in Cancer

### Digestive System Tumors

#### Gastric Cancer

Gastric cancer (GC) is one of the most common cancers causing cancer mortality, especially in Asian populations, and poses a serious threat to human life. Recent clinical data confirmed that miR-484 was significantly downregulated in GC tissues compared to paraneoplastic tissues, and similarly, GC cell lines showed a typical decrease in miR-484 expression (35, 104). Surprisingly, GC tissue miR- 484 low levels were closely associated with hypodifferentiated or undifferentiated cells, distant metastasis in lymph nodes, and reduced 5-year overall survival (104). Overexpression of miR-484 significantly reduced subcutaneous tumorigenicity in mouse GC cells (35). Mechanistically, exogenous increase in miR-484 level expression in GC cell lines promoted cell cycle G1 phase arrest and apoptosis through. In addition, it has been proposed that miR-484 directly targets and negatively regulates CCL-18 expression in GC tissues and overexpression of CCL-18 restores the proliferative effect of miR-484 on GC cells (35) **(** **Table 3** **)**. Knockdown of miR-484 and overexpression of CCL-18 both promoted the phosphorylation of PI3K and AKT in MGC-803 cell, suggesting that miR-484 exerts anti-cancer effects by targeting the CCL-18-PI3K/AKT pathway (35). In conclusion, miR-484 as a tumor suppressor can be used as an independent prognostic indicator for gastric cancer patients.

**Table 3:** The role of miR-484 in Cancerous Diseases.

	Disease	Species and tissue or cell type	Alteration of miR-484 expression	Method for miR-484detection	Target gene	Method for target validation	Biological function	Sample size in clinical studies	Ref
1	GC	Human gastric tissue (China); Gastric cell lines (HGC-27, SNU-1, AGS, NCI-N87, GES-1)	down-regulation	qRT-PCR	N/A	N/A	Inhibit cell proliferation, migration, and invasion	The paired GC tissue and matched adjacent normal tissue specimens(n=124)	(104)
2	GC	Human gastric tissue (China); Gastric cell lines (MGC-803, BGC-823, SGC-7901, MKN-45, MKN-7, GES-1)	down-regulation	qRT–PCR	CCL-18	qRT-PCR, WB and Luciferase assays	Inhibit cell proliferation	The GC tissues and paracancerous tissues	(35)
3	GC	Human gastric tissue (Iran);	down-regulation	polyA-qPCR	N/A	N/A	N/A	The GC(n=40), NG (n=31) and GD(n=45) samples	(100)
4	CRC	Human CRC cell lines (HCT116, SW480, SW620, HT-29 and LOVO)	down-regulation	qRT–PCR	N/A	N/A	Suppress cell proliferation and invasion	N/A	(28)
5	CRC	Humam CRC tissues (China) Human CRC cell lines (HCT116, SW480, SW620, HT-29 and LOVO)	down-regulation	qRT–PCR	N/A	N/A	Suppress cell proliferation and invasion	The CRC tissues and adjacent non-tumor tissues (n = 49)	(36)
6	CRC	Humam CRC tissues (China) Human colon epithelium cell line (FHC) Human CRC cell lines (HCT116, SW480, SW620, DLD-1, HT-29)	down-regulation	qRT–PCR	KLF12	qRT-PCR, WB and Luciferase assays	Suppress cell proliferation and invasion	CRC tissues and matched adjacent normal tissues(n=63)	(37)
7	MSI-CRC	Humam CRC tissues (China) MSS cell lines (HT29, Caco2, SW620, and SW480) and MSI cell lines (HCT116, LoVo, and LS174T)	down-regulation	miRCURY LNA microRNA array (version 8.1) qRT–PCR	CD137L	qRT-PCR, WB and Luciferase assays	Tumour suppressor; Arrests the production of IL-8	Divided the CRC specimens into a testing set of 54 samples and a validation set of 67 samples	(33)
8	CRC	Human BP(China)	In stage I-II CRC: down-regulation In stage III-IV: up-regulation	qRT–PCR	N/A	N/A	Contribute to early diagnosis and surveillance of the progress of CRC.	The blood samples of CRC patients of I-IV stage (n=53) and controlled healthy people(n=50)	(105)
9	HCC	Human HCC tissues (China) Human HCC cell line PLC/PRF/5, SK-Hep-1, Hep3B, HepG2	up-regulation	Luciferase reporter	N/A	N/A	Promotes tumorigenesis	HCC primary tumor samples (n = 112) and matched peritumor tissue samples (n = 36), adjacent normal tissue of hepatic hemangioma patients (n = 10)	(38)
10	HCC	Human HCC tissues (China) The human HCC cell lines Hep3B and HCCLM3	up-regulation	qRT–PCR	TUSC5	IHC and WB	Promotes tumorigenesis	Paired HCC and adjacent normal tissue(n=50)	(39)
11	HCC(HGDN)	Human HGDN specimen (China) Mouse liver tissue Cell lines: THLE-3, NIH/3T3, HL7702, QSG7701, human-induced hepatocytes (hiHeps), mouse-induced hepatocytes (miHeps).	up-regulation	H&E staining and in situ hybridisation	SAMD9 TBL1X	TargetScan analysis pull-down assay Luciferase reporter Immunoblot analysis Western blot assays	Induce hepatocellular malignant transformation	All HGDN samples obtained during liver transplantations or liver resections.	(40)
12	PC	Pancreatic ductal adenocarcinoma cell lines: PANC-1, SW1990, CFPAC-1, bxpc-3	down-regulation	DIANA tools and TargetScan	YAP	WB Luciferase reporter qRT-PCR	Suppress cell proliferation Predicts a good outcome in patients with PDAC	N/A	(30)
13	PC	N/A	down-regulation	TCGA database GEO database	N/A	N/A	N/A	N/A	(106)
14	PC	Human BPs (American)	up-regulation	qRT–PCR (normalized using miR-16) TaqMan Array	N/A	N/A	N/A	Serum samples from PC patients (n=19; stage I: 3; stage II: 16) chronic pancreatitis (n = 10), healthy controls (n = 10), and patients with PNETs (n = 10)	(107)
15	PCa	Human prostate cell lines: RWPE-1, RWPE-2,22Rv1, LNCaP, DU145 and PC-3	up-regulation	qRT–PCR	PSMG1	qRT–PCR Luciferase reporter	Promotes recurrence and migration	N/A	(41)
16	PCa	Human BP(China)	Dwon-regulation	qRT–PCR	N/A	N/A	Assess drug therapy	Healthy Males(n=34) PCa Patients (n=72)	(108)
17	PCa	Human urine (Egypt)	Down-regulation	qRT–PCR	N/A	N/A	Predict the occurrence, progression, and prognosis	Healthy Males(n=10) Pca Patients (n=8) Benign Prostatic Hyperplasia Patients(n=12)	(109)
18	RCC (ccRCC)	Human kidney tissue (China) Human ccRCC cell lines (786-O, A498, ACHN and aki-1)	Down-regulation	qRT–PCR	ZEB1	TargetScan database The dual-luciferase reporter experiment qRT-PCR WB	Tumor-Suppressive	The ccRCC samples and matching normal kidney tissue samples(n=40)	(27)
19	RCC (mRCC)	Human kidney tissue (Czech Republic)	Responders to sunitinib: Down-regulation Nonresponders to sunitinib: Up-regulation	High-throughput miRNA analysis qRT–PCR	N/A	N/A	Connected with sunitinib resistance and failure of the mRCC	The mRCC patients treated with sunitinib after 9 months and divided into two groups: (a) responders to the treatment(n=44) (b) nonresponders with rapid progression(n=19).	(110)
20	RCC (mRCC)	Human kidney tissue (Spain)	Responders to sunitinib: Down-regulation Nonresponders to sunitinib: Up-regulation	qRT–PCR	N/A	N/A	Connected with sunitinib resistance and failure of the mRCC	The mRCC patients treated with sunitinib. Responders to the treatment(n=14) Nonresponders with rapid progression(n=6).	(111)
21	LC (NSCLC)	Human BPs (China) NSCLC cell lines: A549, NCI-H460, 95D, H358, 16HBE.	Up-regulation	qRT–PCR	N/A	N/A	Promotes tumorigenesis	serum samples from patients with NSCLC(n=150) and healthy volunteers(n=50)	(112)
22	LC (NSCLC)	Human lung tissue (China) Human NSCLC cell lines, A549, H1650, PC9 and BEAS–2 B	Up-regulation	High-throughput miRNA analysis qRT–PCR	Apaf-1	IF qRT–PCR WB	Promotes tumorigenesis	NSCLC tissues and their matched adjacent non-tumor tissues(n=20)	(42)
23	LC (LUDA)	Exosome from Human BP(China)	Up-regulation	qRT–PCR	N/A	N/A	Promotes tumorigenesis	Human BP exosome from LUAD patients((n=6)) and healthy controls(n=6)	(113)
24	MPM	Human pleural tissue (Turkey)	Up-regulation	qRT–PCR	N/A	N/A	Potential biomarkers	Pleural specimens from MPM patients(n=18) and BAPE patients(n=6)	(114)
25	NPC	Human nasopharyngeal cell line, HONE1	NPC radioresistant patients: Up-regulation	GSE4850 qRT–PCR	OLA1	qRT–PCR mirDIP database	Potential biomarkers	N/A	(43)
26	NPC (NMC)	Human tumors tissue from nasal cavity and maxillary sinus.	Up-regulation	qRT–PCR	N/A	N/A	Potential biomarkers	The tumors of the nasal cavity(n=2) and the maxillary sinus(n=1).	(115)
27	BCa (ER positive)	Human breast carcinoma cell lines (MCF-7 and T-47D)	Down-regulation	qRT–PCR	KLF4	TCGA qRT–PCR WB The dual-luciferase reporter experiment	Reduce tamoxifen resistance	N/A	(44)
28	BCa	Human breast cancer tissues (China) Human breast cancer cell lines (MCF7, MDA-MB-231 (MDA-231), MDA-MB-436 (MDA-436), MDA-MB-468 (MDA-468), ZR-75-30, and Hs-578T)	Up-regulation	qRT–PCR	CDA	qRT–PCR WB The dual-luciferase reporter experiment	Improves chemosensitization and cell proliferation	Primary breast cancer samples(n=193) and noncancerous mammary controls(n=36)	(116)
29	CC	Human cervical cancer tissue (China) and cell lines(S12)	No treatment: Down-regulation 5-Aza-CdR treatment: Up-regulation	qRT–PCR The luciferase reporter assay Genomic bisulfite sequencing	MMP14 HNF1A	qRT–PCR WB The dual-luciferase reporter experiment	Inhibited cell adhesion and tumor growth	The cervical cancer tissues (n=20 pairs)	(32)
30	CC	Human cervical cancer tissues (China) and cell lines: HeLa, Caski, ME-180, C33A, SiHa and SW756	Down-regulation	qRT–PCR	ZEB1 SMAD2	qRT–PCR WB	Inhibits cell growth, cell cycle but exacerbates apoptosis	Human cervical cancer tissues and the adjacent noncancerous tissues(n=15)	(45)
31	CC	Human BPs (China)	Up-regulation	qRT–PCR	N/A	N/A	Differential miRNAs	Venous blood from cervical cancer patients(n=13), CINIII patient(n=1) and normal controls(n=10)	(117)
32	OC	Human BPs (Denmark)	Up-regulation	qRT–PCR	N/A	N/A	Differential miRNAs	The plasma samples from age-matched patients with malignant (n=95) and benign pelvic mass (n =95)	(118)
33	OC	Human BP exosomes (China)	Down-regulation	qRT-PCR	N/A	N/A	Differential miRNAs	The blood samples from OC patients(n=113) and healthy volunteers(n=60)	(119)
34	FTC	Human thyroid tissues (Germany)	Down-regulation	miRNA sequencing	N/A	N/A	Differential miRNAs	FTC samples(n=19) and FA samples(n=23)	(120)
35	Glioma	Human glioma tissues (China) Human glioma cell lines: U87 and U251	Up-regulation	ISH qRT-PCR	MAP2	qRT–PCR WB The dual-luciferase reporter experiment	Augment the tumor-initiating capability	The glioma tissues(n=153) and para-carcinoma tissues(n=30)	(47)
36	OS	Human BPs (China)	Down-regulation	RT-qPCR	N/A	N/A	Differential miRNAs	The BP samples from healthy volunteers, OS patients, and periostitis patients.	(121)

NG, normal gastric tissue; GD, gastric dysplasia; PDAC, pancreas ductal adenocarcinoma; PNET, pancreatic neuroendocrine tumors; ccRCC, clear cell renal cell carcinoma; mRCC, metastatic renal cell carcinoma; NSCLC, non-small cell lung cancer; LUAD, lung adenocarcinomas; MPM, malignant pleural mesothelioma; NPC, nasopharyngeal carcinoma; NMC, nuclear protein of the testis (NUT) midline carcinoma; CIN, cervical intraepithelial neoplasia; FTC, follicular thyroid carcinoma; FA, follicular thyroid adenoma; IF, immunofluorescence; ISH, in situ hybridization; WB, western blotting.

NA, Not answered.

#### Colorectal Cancer

Colorectal cancer (CRC) is the second leading cause of cancer mortality in Western countries. Chai et al. (122) found significant downregulation of miR-484 expression in 20 human CRC tissue samples. In recent years, there is increasing evidence that lncRNAs are involved in the development of CRC by regulating miR-484 expression in a “sponge” manner. For instance, upregulation of lncRNA PGM5-AS1 significantly inhibited miR-484 to promote CRC metastasis (28) **(** **Table 3** **)**. Similarly, LncRNA ZFAS1, a known oncogenic molecule of CRC, is involved in tumorigenesis by targeting miR-484, however miR-484 overexpression reversed the tumor enhancing effect of ZFAS1 on CRC cells (36) **(** **Table 3** **)**. LINC00239 competitively inhibits miR-484 expression and enhances KLF12 expression to promote oncogenicity in colorectal cancer (77). Another study demonstrated by network modeling that the KCNQ1OT1/miR-484/ANKRD36 axis is involved in colon carcinogenesis (123). Interestingly, the drop of miR-484 in microsatellite instable colorectal cancer (MSI-CRC) was associated with CpG island methylation **(** **Table 3** **)**. After demonstrating through *in vivo* and *in vitro* experiments that miR-484 inhibits the expression of CD137L and IL-8, which in turn inhibits the activity of MSI CRC cells (33). The above studies demonstrated that lncRNA-miR-484 is closely related to the development of CRC and is expected to be an anti-cancer target. Surprisingly, miR-484 was not only aberrantly expressed in colon cancer tissues, but also plasma expression levels correlated with colon cancer progression. A study revealed (105) that serum miR-484 expression levels were significantly lower in patients with early-stage CRC (stages I-II) than in healthy controls, whereas serum miR-484 was abnormally elevated in patients with advanced CRC (stages III-IV), suggesting that serum miR-484 could help in early diagnosis and monitoring of CRC progression.

#### Hepatocellular Carcinoma

Hepatocellular Carcinoma (HCC) are the most common substantial tumors worldwide and the second most common cause of cancer-related death. A growing body of literature reports a close relationship between miR-484 and hepatocarcinogenesis development. Wang (39) and Yang (40) et al. found that 62% and 88% of clinical HCC tissue specimens showed significant upregulation of miR-484, respectively. Surprisingly, this phenomenon was frequently accompanied by elevated serum ALT levels (P = 0.024), increased tumor volume (P = 0.010), and elevated T stage (P = 0.001), and importantly, Kaplan-Meier survival analysis showed that patients in the miR-484 high expression group had a significantly shorter survival time than the low expression group (38). Thus, it is evident that miR -484 high expression significantly promoted the progression of HCC. Mechanistically, miR-484 directly targets the tumor suppressor TUSC5 to promote HCC cell proliferation and metastasis (39). In addition, overexpression of miR-484 was able to reverse the tumor suppressive effect of circADAMTS13 during HCC progression (38)**(** **Table 3** **)**.

On the other hand, miR-484 also plays a highly critical driving role in hepatic precancerous lesions. Liver nodules may encounter low grade dysplastic nodules (LGDN), described as non-malignant, and high-grade dysplastic nodules (HGDN), considered as precancerous lesions, preceding progression to HCC. Yang et al. (124)showed that precancerous lesions were accompanied by abnormally high miR-484 expression in a mouse model of HCC constructed using diethylnitrosamine-injected oncogenes. Interestingly, NAFLD and NASH, as a pre-stage of high lipid-induced HCC development, exhibit high expression of miR-484 in serum and liver tissues, which is consistent with our previous study (125). Yang et al. demonstrated that miR-484 tumor-promoting effects are associated with direct negative regulation of Sterile Alpha Motif Domain Containing 9 (SAMD9), an endosome fusion facilitator, and TBL1X (Transducin β-Like 1X-Linked), a proteasome degrader. Nevertheless, only SAMD9 was the true functional target gene of miR-484 for promoting malignant transformation of hepatocytes. Further in-depth studies revealed (40) that miR-484 upregulation in the precancerous state is dependent on TGF-β/Gli and type I IFN pathway activation, which subsequently generates an inflammatory environment conducive to liver tumor development and induces malignant cell transformation and even tumor progression **(** **Table 3** **)**. More importantly, specific acetylation of H3K27 is critical in IFN-induced miR-484 transcriptional activation and cellular transformation (40). Convincingly, the tumorigenic effect of mmu-miR-484 was substantially attenuated in TGF-β and IFN-β knockout mice (40), suggesting that miR-484 may induce tumorigenesis through pro-inflammation. Hence, miR-484 inhibitors may be an option for hepatocellular carcinoma treatment in the future. Ultimately, plasma miR-484 is significantly upregulated in HCV-mediated HCC and cirrhosis (76), so miR-484 has promising potential as a biomarker for disease diagnosis in addition to its therapeutic potential.

#### Pancreatic Cancer

Given that pancreatic cancer (PC) is characterized by difficult early diagnosis, low surgical resection rate, easy recurrence and metastasis after surgery, and very poor prognosis, finding more effective methods for early detection of pancreatic cancer is an urgent need. In both clinical PC specimens and pancreatic ductal adenocarcinoma cell lines, high levels of consistent lncRNA THAP9-AS1 expression were observed, which competitively inhibited the negative regulatory effect of miR-484 on YAP and thus accelerated the proliferation of pancreatic ductal adenocarcinoma cells (30). Moreover, miR-484 directly induced downregulation of YAP1 expression involved in apoptosis and inflammatory response in cardiomyocytes (34) **(** **Table 3** **)**. Similarly, Ma et al. (106) identified 19 differentially expressed miRNAs (DE-miRNAs) with down-regulated expression, including miR-484, by integrating data from the Cancer Genome Atlas and Gene Expression Comprehensive Database. Traditionally, the expression levels of miRNAs in tissues and serum have been significantly correlated. However, another study found that circulating miR-484 levels were significantly higher in pancreatic cancer patients compared to controls (107). miR-484 expression trends in tissues and serum were contrasting. It is known that miRNA abundance is much lower compared to tissues, but has an abnormal stability unmatched by tissues. Since only 19 PC patient samples were selected in the latter experimental design, which may cause sample bias, the sample size needs to be expanded to further clarify the altered expression of serum miR-484. In conclusion, miR-484, as a key tumor suppressor gene and miRNA related to the pathogenesis of pancreatic cancer, is expected to improve the early detection of pancreatic cancer in the future.

### Urological Tumors

#### Prostate Cancer

Prostate cancer (PCa) has become one of the fastest growing male malignancies in the last decade. Surprisingly, miR-484 plays a role as a candidate oncogene in the development, progression and recurrence of PCa. Lee et al. (41) first demonstrated that miR-484 was significantly negatively associated with disease-free survival and highly expressed in PCa tissues. Upregulation of miR-484 expression in prostate tumors is associated with early disease recurrence. Mechanistically, miR-484 directly targets PSMG1 to enhance the invasiveness of cancer cells.

Further, a Meta-analysis of multiple miRNA datasets on existing recurrent PCa found that miR-484 is one of the commonly upregulated miRNAs (126). This is consistent with the former result. In recent years, early sensitive serological markers regarding PCa have been the focus of clinical studies. It has been demonstrated that miR-1825 and miR-484 are highly specific in PCa patients’ sera (108) **(** **Table 3** **)**. Interestingly, miRNA expression profiling by collecting urine from PCa patients, benign prostatic hyperplasia (BPH) patients and healthy men revealed that miR-484 had a high sensitivity (80%) for detecting PCa, while miR-1825/miR-484 combination(75%) was able to enhance the specificity of miR-484 alone(19%) for detecting PCa in BPH individuals (109). Consequently, monitoring changes in serum and urine miR-484 expression levels can help in PCa risk assessment and therapeutic intervention.

#### Renal Cell Carcinoma

The lncRNA PCED1B-AS1 located on human chromosome 12q13.11 targets and regulates miR-484 to play an oncogenic role in colorectal tumorigenesis, as described previously (28). Notably, PCED1B-AS1 and miR-484 are synergistically involved in the development of clear renal cell carcinoma (ccRCC). ccRCC tissues showing high expression of PCED1B-AS1 and low expression of miR-484 are commonly associated with high tumor stage, high Fuhrman grading, and shortened overall patient survival (127). The dual luciferase assay validated the direct targeting relationship (127) **(** **Table 3** **)**. Overexpression of miR-484 exacerbated the negative regulation of the downstream target ZEB1 and thus reversed PCED1B-AS1-induced proliferation and migration of ccRCC cells. Remarkably, besides miR-484, PCED1B-AS1 may also target multiple miRNAs that may be involved in the regulation of multiple signaling pathways.

Currently, the tyrosine kinase inhibitor sunitinib has become the first-line agent for the treatment of metastatic renal cell carcinoma. Based on the current clinical patient response to sunitinib-induced drug resistance and the rate of disease progression are both heterogeneous. Several studies have sought biomarker miRNAs that predict response to sunitinib treatment by detecting miRNA changes in metastatic renal cell carcinoma after sunitinib treatment. Patients in the sunitinib-treated group with reduced levels of miR-155 and miR-484 were able to prolong the time to progression (TTP) (110). Consistent with the former results, another study found that high miR-484 expression was significantly associated with reduced TTP and overall survival by comparing tumor tissues from patients with extreme phenotypes of mRCC with significant efficacy and resistance to sunitinib (111). Combining the above data confirmed that miR-484 expression differences in mRCC patients were closely related to significant sensitivity or resistance to sunitinib.

### Respiratory Tumors

#### Lung Cancer

Elevated serum miR-484 has been reported to be positively correlated with histologic grade, lymph node metastasis, distant metastasis and clinical stage in NSCLC patients, but there was no statistically significant relationship between patient gender, age or tumor volume (112). It was also noted that miR-484 upregulation significantly decreased the overall survival rate of NSCLC patients (112). Further mechanistic studies confirmed that miR-484 knockdown induced cell cycle arrest and thus inhibited cell growth (112). These results suggest that serum miR-484 may serve as a potential noninvasive biomarker for NSCLC. Likewise, another study found that miR-484 expression was significantly higher in NSCLC tissues than in matched adjacent non-cancerous tissues (42). Convincingly, *in vivo* experiments in mice demonstrated that miR-484 overexpression significantly increased tumor volume (113). miR-484 was mechanistically found to enhance migration and proliferation of NSCLC through inhibition of Apaf-1 and caspase-3 expression (113) **(** **Table 3** **)**.

Exosomes serve as an important pathway for material and signaling communication between cells. In recent years, miRNAs contained within exosomes have had a major boost in the diagnosis and treatment of tumor diseases. Xue et al. (113) first analyzed and compared plasma exosomal miRNA expression differences in patients with lung adenocarcinoma (LUAD) and performed Kaplan-Meier survival analysis based on online clinical data and found that patients with high miR-484 expression generally had lower survival rates. It was concluded that exosomal miR-484 is a potential prognostic marker for LUAD. Overall, miR-484 plays an oncosuppressor role in lung malignancies.

### Breast Cancer

Breast cancer (BCa) is the most common malignant tumor in women and its incidence has been on a continuous increase in the last decade. Given that the breast is an estrogen-dependent organ, patients with estrogen receptor (ER)-positive BCa may benefit from endocrine therapy that targets the ER pathway. Nevertheless, the development of resistance to endocrine therapy remains a major clinical concern. The engagement of miR-484 in studies related to sunitinib resistance in metastatic renal cancer has been previously reported in the literature (110, 111, 128). Wei et al. (44) showed that tamoxifen-resistant BCa cells showed a malignant phenotype with high KLF4 expression, increased stemness and invasiveness. It is notable that miR-484 directly negatively regulates KLF4 expression to induce re-sensitization of BCa cells to tamoxifen (116). Furthermore, chemotherapy is crucial for patients with advanced BCa, whether ER-positive, ER-negative, or human epidermal growth factor receptor 2 (HER2)-positive. Currently, gemcitabine is unanimously recommended by national and international guidelines for the treatment of advanced BCa. Studies have confirmed that cytidine nucleoside deaminase (CDA) is significantly upregulated in a gemcitabine-resistant BCa model and plays a key role in regulating cell cycle redistribution and replication (116) **(** **Table 3** **)**. Notably, miR-484 was upregulated in primary BCa tissues compared to CDA. Further studies confirmed that overexpression of miR-484 in BCa cells markedly inhibited CDA-mediated gemcitabine resistance, cell proliferation regulation and cell cycle redistribution, thereby enhancing gemcitabine sensitivity. In conclusion, miR-484 has some potential in resisting chemotherapeutic drug resistance. Beyond this, miR-484 is worthy of attention in terms of breast cancer disease and prognosis. Shi et al. (129) concluded from a comparative analysis of miRNA profiles in 253 patients with invasive BCa that higher hsa-miR-484 expression was associated with worse prognosis **(** **Table 3** **)**. Similarly, Volinia et al. (130) by integrating miRNA sequencing data from 466 patients with primary invasive ductal carcinoma (IDC) and performing survival analysis similarly found a strong association between miR-484 and prognosis of IDC patients **(** **Table 3** **)**.

Indeed, differential expression of miR-484 was also present in the sera of Bca patients. Elevated miR-484 levels were found in breast cancer sera compared to healthy volunteers (131, 132). However, serum miR-484 levels did not correlate with pathological grade and tumor size (131). Such results are based on 39 early-stage breast cancer patients with serum indicators and may lead to unexpected and surprising findings if the sample is further expanded or if more subjects with different stages of Bca are included. In conclusion, miR-484 may be demonstrated and applied to the clinical diagnosis, treatment and prognosis of breast cancer.

### Cervical Cancer

Recently miRNAs may help to improve the early detection of malignancies in women. A latest study (117) revealed that circulating miR-484 has potential in identifying cervical cancer (CC) and cervical intraepithelial neoplastic cycle (CIN) and is expected to be a non-invasive biomarker for CC. Previously, Andrea Ritter et al. (133) suggested that miR-484/-23a in serum could be a potential diagnostic marker for CC. Tumor or lesion cells can secrete aberrant miRNAs into the blood. Thus, dysregulated miRNAs identified from patient plasma may influence tumor or lesion cytogenesis. In combination with the published data to date, it is confirmed that miR-484 plays an anticancer role in CC histogenesis and cell development.

A research uncovered that reduced miR-484 expression levels in clinical cervical cancer specimens and cell lines could restore ZEB1 and SMAD2, key transcription factors of epithelial-mesenchymal transition (EMT), inducing CC malignant behavior (45) **(** **Table 3** **)**. Additionally, Hu et al. (32) revealed for the first time the presence of a CpG island in the miR-484 promoter region (-218 to +5). Both bisulfite genome sequencing and luciferase reporter gene analysis confirmed that miR-484 promoter activity and expression levels were lower in CC than in normal cervical epithelial cells, which was attributed to the direct induction of miR-484 promoter CpG hypermethylation by EZH2-mediated DNA methyltransferase 1 (DNMT1) **(** **Table 3** **).** Further studies indicated that miR-484 exerts anti-cancer effects by inhibiting the MMP14-TNF axis and HNF1A-Wnt axis involved in CC cell adhesion, migration, invasion and EMT expression (32) **(** **Table 3** **)**. This is consistent with the former results. miR-484 acts as a key regulator of CC metastasis inhibition, extending our understanding of the molecular mechanisms underlying CC progression and metastasis.

### Ovarian Cancer

Ovarian cancer (OC) is currently regarded as a “silent killer” of women’s life and health, due to its late diagnosis and high recurrence rate. Although the combination of platinum and paclitaxel analogs is the first-line regimen for treating patients with ovarian cancer, chemoresistance is a key factor constraining the improvement of ovarian cancer cure rates. miR-484 was found to be closely associated in OC biology and chemoresistance by Andrea Vecchione et al. (46) through the analysis of 198 ovarian plasmacytoma tissue specimens. VEGFB- VEGFR1 axis is recognized as an important signaling pathway involved in OC neoangiogenesis. Interestingly, miR-484 in OC cells directly regulates VEGFB protein on tumor cells or inhibits the receptor VEGFR2 targeting VEGF signaling pathway on tumor-associated endothelial cells in a paracrine manner to control angiogenesis, leading to normalization of tumor microenvironment and enhanced drug sensitivity (46) **(** **Table 3** **)**.

The late diagnosis of OC is partly due to the absence of reliable non-invasive tests to help early identification of the pathological nature of the tumor. Free cell or plasma miRNAs have been shown to be effective in both normal and cancerous tissue classification and cancer prognosis. Plasma miR-484 has potential in differentiating benign from malignant ovarian tumors. miR-484 was significantly upregulated in serum of OC patients (118). Yet another study (119) detected a dramatic decrease in serum exosome miR-484 levels in OC patients, accompanied by an exacerbation of the malignant phenotype and a reduction in overall and progression-free survival. The above two reports on circulating miR-484 expression in OC showed distinctly opposite results. Notably, the composition of miRNAs and their abundance in plasma exosomes is different from that in the overall blood. Some miRNA differential expression is evident in exosomes, but miRNA expression in overall blood is masked by other complex and diverse molecules. Moreover, miRNAs were detected in exosomes and plasma samples with divergent changes, implying that distinct mechanisms are used to regulate miRNA packaging in exosomes or other vectors (134). Since there are relatively few studies related to the involvement of miR-484 in OC development, whether miR-484 plays a pro-oncogenic role, an oncogenic role or a dual role in OC is unclear. In spite of the fact that the mechanism is not yet clear, it is undeniable that the sensitizing effect of miR-484 on chemotherapeutic drugs may hold promise for OC patients in the future.

### Other Tumor Diseases

The circulating two-miRNA classifier (miR-484/miR-148b-3p) has a degree of advantage in differentiating thyroid tumors (sensitivity of 89% and specificity of 87%) (120). However still further validation in histology is needed to better compensate and improve the clinical uncertainty of mutation-negative fine needle aspiration test. In addition, low miR-484 expression in serum of patients with osteosarcoma is closely associated with clinical malignant phenotype, poor prognosis, and has been initially confirmed as a promising biomarker (121).

Yi et al. (47) unveiled that the combination of low miR-484 and high MAP2 levels is associated with the best prognosis of glioma. Further in-depth exploration revealed that miR-484 promotes tumor initiation properties by activating ERK1/2 and Myc signaling through MAP2-mediated interaction between Grb2 and SOS **(** **Table 3** **)**. Interestingly, miR-484 is regulated by c-Myc in glioma cells, so that c-myc-miR-484- ERK1/2 constitutes a closed positive feedback loop exacerbating the gliomagenesis process. Notably, the stem cell nature of tumors is closely related to the resistance to tumor therapy. Therefore, it remains to be further confirmed whether miR-484 occurs by the same mechanism in different types of tumors presenting a high degree of chemotherapy insensitivity.

A recent study revealed a significant upregulation of hsa-miR-484 in pleural tissue of patients with malignant pleural mesothelioma (MPM) compared to benign asbestos-associated pleural effusion (BAPE), indicating that miR-484 could be a new potential biomarker for the diagnosis of mesothelioma (114). Meanwhile, Zhao et al. (43) searched and analyzed publicly available microarray data and found that miR-484 was remarkably up-regulated in radiation-resistant nasopharyngeal carcinoma (NPC) samples accompanied by decreased expression of miR-484-targeted genes and verified the negative regulation of miR-484 with OLA1 by qRT-PCR method **(** **Table 3** **)**. Similarly, miR-484 was also upregulated by RT-qPCR in NUT midline carcinoma (NMC) specimens located in the nasal cavity and maxillary sinus (broadly classified as NPC) (115) **(** **Table 3** **)**. Although the mechanism of miR-484 expression upregulation is unclear, these data may provide a basis for future studies on the molecular mechanisms of treatment and radiation resistance in NPC.

## Perspective

**Figure 3:**
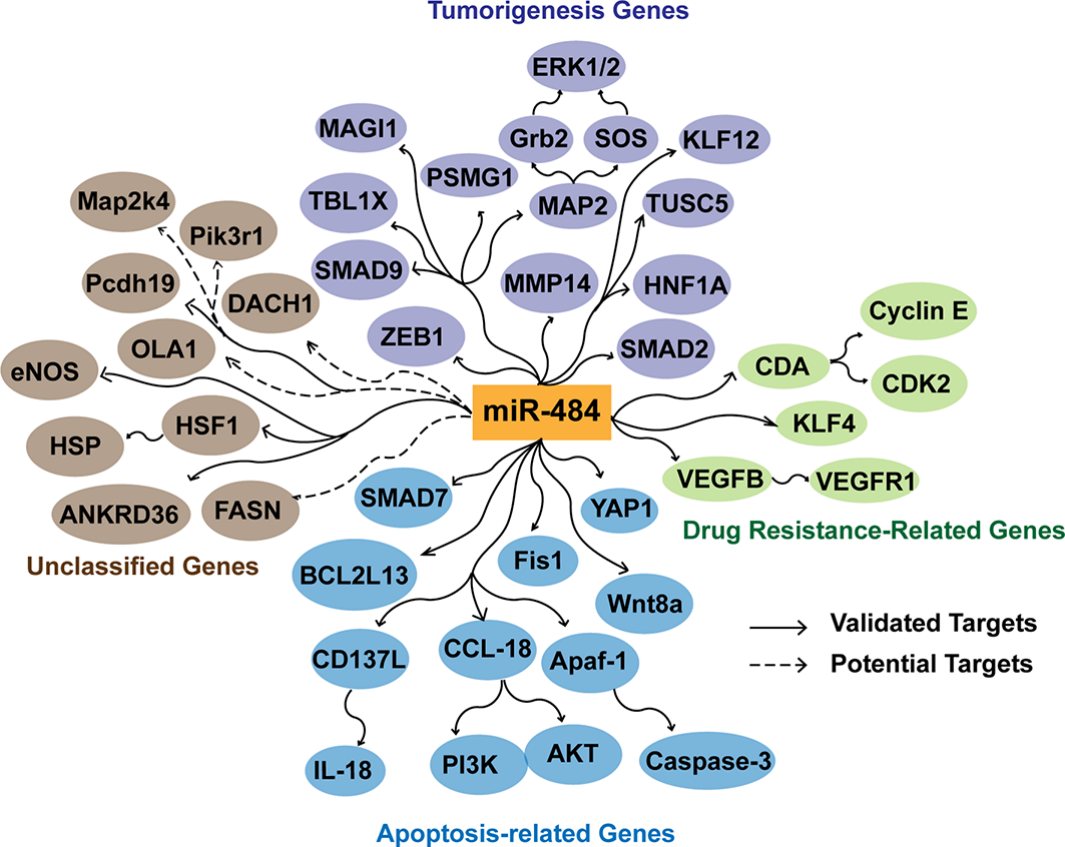
Effects of miR-484 target genes in apoptosis, tumorigenesis, and tumor drug resistance. miR-484 inhibits the tumorigenic process by suppressing the expression of ZEB1, SMAD2, HNF1A, MMP14, MAP2, PSMG1, SMAD9, MAGI1, and TBL1X. miR-484 targets SMAD7, YAP1, Fis1 BCL2L13, CD137L, Apaf-1, CCL18 and Wnt8a to affect the level of apoptosis. In addition, miR-484 regulates chemoresistance of cancer cells by targeting CDA, KLF-4, VEGFB and VEGFR1. Potential targets are screened and predicted through a database.

**Figure 4:**
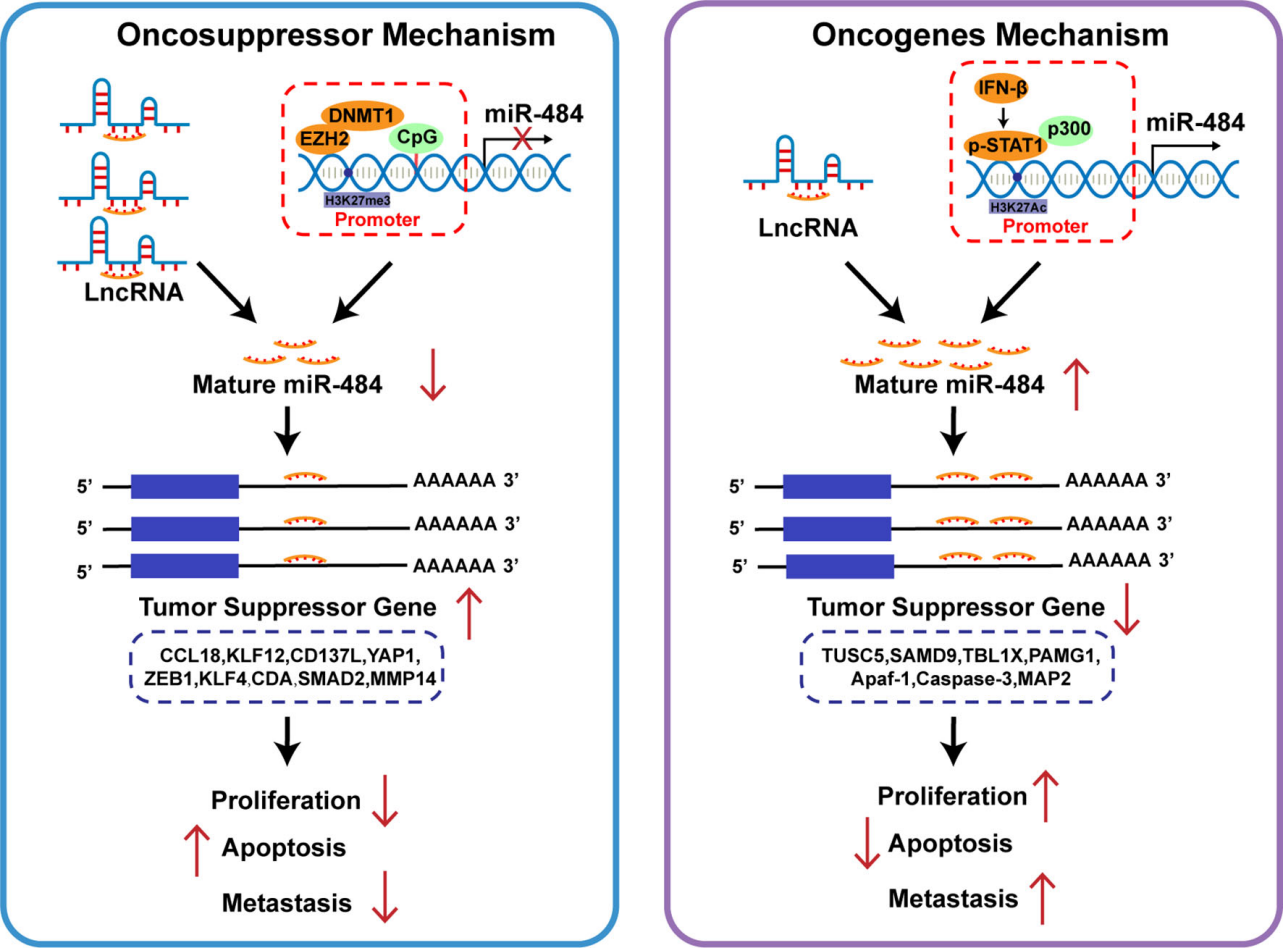
Role of miR-484 and its target genes on cancer cell biology. Different cell types or tissues regulate miR-484 mainly by LncRNA competitive repression and epigenetic mechanisms. miR-484 exerts oncogenic or pro-carcinogenic effects in different cancers by targeting genes related to cell proliferation and apoptosis.

In recent years miRNAs have greatly enriched the clinical understanding of diseases, including tumors, as key regulators of different human diseases. Based on the data reported so far, it is clear that miR-484 is expressed in multiple types of cells and tissues and targets multiple mRNAs. In this review, the development of many diseases accompanied by miR-484 overexpression or inhibition is to some extent predictive of disease outcome. It is noteworthy that the way of regulating miR-484 expression and miR-484 targeting regulatory molecules are different in different types of tissues or even different injury factors in the same tissues **(** **Figures 3, 4**). The reasons for differential miR-484 expression, however, could be mutations in miR-484 seed sequences, altered promoter methylation levels, blocked miR-484 maturation processes and competitive repression by accepting non-partial lncRNAs, among others **(** **Figure 4** **)**. Moreover, miR-484 targets different mRNAs in different cellular settings which may result from differential expression of miR-484 basal or competition with RNA-binding proteins. To our surprise, miR-484 directly targets amino acid coding sequences that block the translation process of proteins such as Fis1.

We outlined some of the biological functions and target genes of miR-484 to elucidate the multiple potential mechanisms of miR-484 **(** **Figure 3** **)**. Considering that most common disease screening methods fail to accurately screen tumors at early stages of disease, detecting miR-484 expression in serum and tissues may become an effective target for tumor diagnosis, prognosis and treatment, providing a promising application for miRNA in tumor therapy. However, many questions remain to be clarified, such as the specific regulatory mechanism of miRNA-484 in tumors **(** **Figure 4** **)**. Given that miR-484 can act as both a proto-oncogene and an oncogene, how to develop precise therapeutic strategies based on the biological properties of miR-484 still needs to be explored. Further research on the above issues will help to explore the mechanism of tumorigenesis and promote the development of tumor-related clinical diagnostic methods and new therapeutic targets. At the same time, the improvement of RNA molecular delivery technology has also made miRNA-based disease treatment options more realistic.

## Author Contributions

Zf-S and Yz-J drafted the manuscript. Zf-S provided funding supports. Yz-J and JL checked the figures and tables and revised the manuscript. Gq-W edited and added the constructive suggestions on the manuscript. JL conducted bioinformatics database analysis. All authors contributed to the article and approved the submitted version.

## Funding

This work was supported by National Natural Science Foundation of China (Grant Nos. 81974040 and 81670374).

## Conflict of Interest

The authors declare that the research was conducted in the absence of any commercial or financial relationships that could be construed as a potential conflict of interest.

## Publisher’s Note

All claims expressed in this article are solely those of the authors and do not necessarily represent those of their affiliated organizations, or those of the publisher, the editors and the reviewers. Any product that may be evaluated in this article, or claim that may be made by its manufacturer, is not guaranteed or endorsed by the publisher.

## References

[1] Eulalio A Huntzinger E Izaurralde E. Getting to the Root of miRNA-Mediated Gene Silencing. Cell (2008) 132(1):9–14. doi: 10.1016/j.cell.2007.12.024 18191211

[2] Avital G Franca GS Yanai I. Bimodal Evolutionary Developmental miRNA Program in Animal Embryogenesis. Mol Biol Evol (2018) 35(3):646–54. doi: 10.1093/molbev/msx316 29237075

[3] Knauss JL Bian S Sun T. Plasmid-Based Target Protectors Allow Specific Blockade of miRNA Silencing Activity in Mammalian Developmental Systems. Front Cell Neurosci (2013) 7:163. doi: 10.3389/fncel.2013.00163 24068984 PMC3781311

[4] Pasquariello R Manzoni E Fiandanese N Viglino A Pocar P Brevini T. Implications of miRNA Expression Pattern in Bovine Oocytes and Follicular Fluids for Developmental Competence. Theriogenology (2020) 145:77–85. doi: 10.1016/j.theriogenology.2020.01.027 32004821

[5] Meganathan K Jagtap S Srinivasan SP Wagh V Hescheler J Hengstler J. Neuronal Developmental Gene and miRNA Signatures Induced by Histone Deacetylase Inhibitors in Human Embryonic Stem Cells. Cell Death Dis (2015) 6:e1756. doi: 10.1038/cddis.2015.121 25950486 PMC4669700

[6] Pallocca G Fabbri M Sacco MG Gribaldo L Pamies D Laurenza I. miRNA Expression Profiling in a Human Stem Cell-Based Model as a Tool for Developmental Neurotoxicity Testing. Cell Biol Toxicol (2013) 29(4):239–57. doi: 10.1007/s10565-013-9250-5 23903816

[7] Fabian MR Sonenberg N. The Mechanics of miRNA-Mediated Gene Silencing: A Look Under the Hood of miRISC. Nat Struct Mol Biol (2012) 19(6):586–93. doi: 10.1038/nsmb.2296 22664986

[8] Matsui M Chu Y Zhang H Gagnon KT Shaikh S Kuchimanchi S. Promoter RNA Links Transcriptional Regulation of Inflammatory Pathway Genes. Nucleic Acids Res (2013) 41(22):10086–109. doi: 10.1093/nar/gkt777 PMC3905862 23999091

[9] Suzuki HI Young RA Sharp PA. Super-Enhancer-Mediated RNA Processing Revealed by Integrative MicroRNA Network Analysis. Cell (2017) 168(6):1000–14. doi: 10.1016/j.cell.2017.02.015 PMC5350633 28283057

[10] Xiao M Li J Li W Wang Y Wu F Xi Y. MicroRNAs Activate Gene Transcription Epigenetically as an Enhancer Trigger. RNA Biol (2017) 14(10):1326–34. doi: 10.1080/15476286.2015.1112487 PMC5711461 26853707

[11] Taverna S Amodeo V Saieva L Russo A Giallombardo M De Leo G. Exosomal Shuttling of miR-126 in Endothelial Cells Modulates Adhesive and Migratory Abilities of Chronic Myelogenous Leukemia Cells. Mol Cancer (2014) 13:169. doi: 10.1186/1476-4598-13-169 25015105 PMC4105877

[12] Calin GA Dumitru CD Shimizu M Bichi R Zupo S Noch E. Frequent Deletions and Down-Regulation of Micro- RNA Genes Mir15 and Mir16 at 13q14 in Chronic Lymphocytic Leukemia. Proc Natl Acad Sci USA (2002) 99(24):15524–9. doi: 10.1073/pnas.242606799 PMC137750 12434020

[13] Weissmann-Brenner A Kushnir M Lithwick YG Aharonov R Gibori H Purim O. Tumor microRNA-29a Expression and the Risk of Recurrence in Stage II Colon Cancer. Int J Oncol (2012) 40(6):2097–103. doi: 10.3892/ijo.2012.1403 22426940

[14] Ngankeu A Ranganathan P Havelange V Nicolet D Volinia S Powell BL. Discovery and Functional Implications of a miR-29b-1/miR-29a Cluster Polymorphism in Acute Myeloid Leukemia. Oncotarget (2018) 9(4):4354–65. doi: 10.18632/oncotarget.23150 PMC5796978 29435107

[15] Liu Z Huang F Luo G Wang Y Du R Sun W. miR-214 Stimulated by IL-17A Regulates Bone Loss in Patients With Ankylosing Spondylitis. Rheumatol (Oxf) (2020) 59(5):1159–69. doi: 10.1093/rheumatology/kez594 31846044

[16] Cheng Y Wang D Wang F Liu J Huang B Baker MA. Endogenous miR-204 Protects the Kidney Against Chronic Injury in Hypertension and Diabetes. J Am Soc Nephrol (2020) 31(7):1539–54. doi: 10.1681/ASN.2019101100 PMC7350998 32487559

[17] Gjorgjieva M Sobolewski C Dolicka D Correia DSM Foti M. miRNAs and NAFLD: From Pathophysiology to Therapy. Gut (2019) 68(11):2065–79. doi: 10.1136/gutjnl-2018-318146 31300518

[18] Gabisonia K Prosdocimo G Aquaro GD Carlucci L Zentilin L Secco I. MicroRNA Therapy Stimulates Uncontrolled Cardiac Repair After Myocardial Infarction in Pigs. Nature (2019) 569(7756):418–22. doi: 10.1038/s41586-019-1191-6 PMC6768803 31068698

[19] Allach EKL Heide S Caberg JH Andrieux J Doco FM Vincent-Delorme C. 16p13.11 Microduplication in 45 New Patients: Refined Clinical Significance and Genotype-Phenotype Correlations. J Med Genet (2020) 57(5):301–7. doi: 10.1136/jmedgenet-2018-105389 30287593

[20] Fujitani M Zhang S Fujiki R Fujihara Y Yamashita T. A Chromosome 16p13.11 Microduplication Causes Hyperactivity Through Dysregulation of miR-484/Protocadherin-19 Signaling. Mol Psychiatry (2017) 22(3):364–74. doi: 10.1038/mp.2016.106 PMC5322274 27378146

[21] Li QL Ju ZH Huang JM Li JB Li RL Hou MH. Two Novel SNPs in HSF1 Gene Are Associated With Thermal Tolerance Traits in Chinese Holstein Cattle. DNA Cell Biol (2011) 30(4):247–54. doi: 10.1089/dna.2010.1133 21189066

[22] Li QL Zhang ZF Xia P Wang YJ Wu ZY Jia YH. A SNP in the 3’-UTR of HSF1 in Dairy Cattle Affects Binding of Target bta-miR-484. Genet Mol Res (2015) 14(4):12746–55. doi: 10.4238/2015.October.19.18 26505425

[23] Wang K Sun T Li N Wang Y Wang JX Zhou LY. MDRL lncRNA Regulates the Processing of miR-484 Primary Transcript by Targeting miR-361. PloS Genet (2014) 10(7):e1004467. doi: 10.1371/journal.pgen.1004467 25057983 PMC4109843

[24] Zhang R Feng Y Lu J Ge Y Li H. lncRNA Ttc3-209 Promotes the Apoptosis of Retinal Ganglion Cells in Retinal Ischemia Reperfusion Injury by Targeting the miR-484/Wnt8a Axis. Invest Ophthalmol Vis Sci (2021) 62(3):13. doi: 10.1167/iovs.62.3.13 PMC7960841 33687475

[25] Liu X Wang X Zhang L Zhou Y Yang L Yang M. By Targeting Apoptosis Facilitator BCL2L13, microRNA miR-484 Alleviates Cerebral Ischemia/Reperfusion Injury-Induced Neuronal Apoptosis in Mice. Bioengineered (2021) 12(1):948–59. doi: 10.1080/21655979.2021.1898134 PMC8806345 33724167

[26] Liu H Li S Jiang W Li Y. MiR-484 Protects Rat Myocardial Cells From Ischemia-Reperfusion Injury by Inhibiting Caspase-3 and Caspase-9 During Apoptosis. Korean Circ J (2020) 50(3):250–63. doi: 10.4070/kcj.2019.0107 PMC7043966 31845557

[27] Qin J Zhu T Wu W Chen H He Y. Long Non-Coding RNA PCED1B-AS1 Promotes the Progression of Clear Cell Renal Cell Carcinoma Through miR-484/ZEB1 Axis. Onco Targets Ther (2021) 14:393–402. doi: 10.2147/OTT.S270149 33469315 PMC7813644

[28] Shen Y Qi L Li Y Zhang Y Gao X Zhu Y. The Downregulation of lncRNA Pgm5-As1 Inhibits the Proliferation and Metastasis *Via* Increasing miR-484 Expression in Colorectal Cancer. Cancer Biother Radiopharm (2021) 36(2):220–9. doi: 10.1089/cbr.2019.3059 32354224

[29] Cao C Li J Li G Hu G Deng Z Huang B. Long Non-Coding RNA TMEM220-AS1 Suppressed Hepatocellular Carcinoma by Regulating the miR-484/MAGI1 Axis as a Competing Endogenous RNA. Front Cell Dev Biol (2021) 9:681529. doi: 10.3389/fcell.2021.681529 34422806 PMC8376477

[30] Li N Yang G Luo L Ling L Wang X Shi L. lncRNA THAP9-AS1 Promotes Pancreatic Ductal Adenocarcinoma Growth and Leads to a Poor Clinical Outcome *via* Sponging miR-484 and Interacting With YAP. Clin Cancer Res (2020) 26(7):1736–48. doi: 10.1158/1078-0432.CCR-19-0674 31831555

[31] Gongol B Marin T Zhang J Wang SC Sun W He M. Shear Stress Regulation of miR-93 and miR-484 Maturation Through Nucleolin. Proc Natl Acad Sci USA (2019) 116(26):12974–9. doi: 10.1073/pnas.1902844116 PMC6600934 31182601

[32] Hu Y Wu F Liu Y Zhao Q Tang H. DNMT1 Recruited by EZH2-Mediated Silencing of miR-484 Contributes to the Malignancy of Cervical Cancer Cells Through MMP14 and HNF1A. Clin Epigenet (2019) 11(1):186. doi: 10.1186/s13148-019-0786-y PMC6898970 31810492

[33] Mei Q Xue G Li X Wu Z Li X Yan H. Methylation-Induced Loss of miR-484 in Microsatellite-Unstable Colorectal Cancer Promotes Both Viability and IL-8 Production *via* CD137L. J Pathol (2015) 236(2):165–74. doi: 10.1002/path.4525 25727216

[34] Xu M Li XY Song L Tao C Fang J Tao L. miR-484 Targeting of Yap1-Induced LPS-Inhibited Proliferation, and Promoted Apoptosis and Inflammation in Cardiomyocyte. Biosci Biotechnol Biochem (2021) 85(2):378–85. doi: 10.1093/bbb/zbaa009 33604630

[35] Liu J Li SM. MiR-484 Suppressed Proliferation, Migration, Invasion and Induced Apoptosis of Gastric Cancer *via* Targeting CCL-18. Int J Exp Pathol (2020) 101(6):203–14. doi: 10.1111/iep.12366 PMC7691219 32985776

[36] Xie S Ge Q Wang X Sun X Kang Y. Long non-Coding RNA ZFAS1 Sponges miR-484 to Promote Cell Proliferation and Invasion in Colorectal Cancer. Cell Cycle (2018) 17(2):154–61. doi: 10.1080/15384101.2017.1407895 PMC5884120 29179614

[37] Luo X Yue M Li C Sun D Wang L. Long Non-Coding RNA LINC00239 Functions as a Competitive Endogenous RNA by Sponging microRNA-484 and Enhancing KLF12 Expression to Promote the Oncogenicity of Colorectal Cancer. Onco Targets Ther (2020) 13:12067–81. doi: 10.2147/OTT.S278582 PMC7695693 33262607

[38] Qiu L Huang Y Li Z Dong X Chen G Xu H. Circular RNA Profiling Identifies Circadamts13 as a miR-484 Sponge Which Suppresses Cell Proliferation in Hepatocellular Carcinoma. Mol Oncol (2019) 13(2):441–55. doi: 10.1002/1878-0261.12424 PMC6360375 30537115

[39] Wang S Wang W Han X Wang Y Ge Y Tan Z. Dysregulation of Mir484-TUSC5 Axis Takes Part in the Progression of Hepatocellular Carcinoma. J Biochem (2019) 166(3):271–9. doi: 10.1093/jb/mvz034 31157375

[40] Yang Y Lin X Lu X Luo G Zeng T Tang J. Interferon-microRNA Signalling Drives Liver Precancerous Lesion Formation and Hepatocarcinogenesis. Gut (2016) 65(7):1186–201. doi: 10.1136/gutjnl-2015-310318 PMC6624432 26860770

[41] Lee D Tang W Dorsey TH Ambs S. miR-484 is Associated With Disease Recurrence and Promotes Migration in Prostate Cancer. Biosci Rep (2020) 40(5). doi: 10.1042/BSR20191028 PMC7953493 32338277

[42] Li T Ding ZL Zheng YL Wang W. MiR-484 Promotes Non-Small-Cell Lung Cancer (NSCLC) Progression Through Inhibiting Apaf-1 Associated With the Suppression of Apoptosis. BioMed Pharmacother (2017) 96:153–64. doi: 10.1016/j.biopha.2017.09.102 28982084

[43] Zhao H Chang A Ling J Zhou W Ye H Zhuo X. Construction and Analysis of miRNA-mRNA Regulatory Networks in the Radioresistance of Nasopharyngeal Carcinoma. 3 Biotech (2020) 10(12):511. doi: 10.1007/s13205-020-02504-x PMC7648832 33184596

[44] Wei Y Li H Qu Q. miR-484 Suppresses Endocrine Therapy-Resistant Cells by Inhibiting KLF4-Induced Cancer Stem Cells in Estrogen Receptor-Positive Cancers. Breast Cancer-Tokyo (2021) 28(1):175–86. doi: 10.1007/s12282-020-01152-6 32865695

[45] Hu Y Xie H Liu Y Liu W Liu M Tang H. miR-484 Suppresses Proliferation and Epithelial-Mesenchymal Transition by Targeting ZEB1 and SMAD2 in Cervical Cancer Cells. Cancer Cell Int (2017) 17:36. doi: 10.1186/s12935-017-0407-9 28286418 PMC5339969

[46] Vecchione A Belletti B Lovat F Volinia S Chiappetta G Giglio S. microRNA Signature Defines Chemoresistance in Ovarian Cancer Through Modulation of Angiogenesis. Proc Natl Acad Sci USA (2013) 110(24):9845–50. doi: 10.1073/pnas.1305472110 PMC3683704 23697367

[47] Yi R Feng J Yang S Huang X Liao Y Hu Z. miR-484/MAP2/c-Myc-Positive Regulatory Loop in Glioma Promotes Tumor-Initiating Properties Through ERK1/2 Signaling. J Mol Histol (2018) 49(2):209–18. doi: 10.1007/s10735-018-9760-9 29480405

[48] Wang K Long B Jiao JQ Wang JX Liu JP Li Q. miR-484 Regulates Mitochondrial Network Through Targeting Fis1. Nat Commun (2012) 3:781. doi: 10.1038/ncomms1770 22510686

[49] Miyazawa R Iso Y Tsujiuchi M Shoji M Takahashi T Koba S. Potential Association of Circulating MicroRNA-181c and MicroRNA-484 Levels With Cardiorespiratory Fitness After Myocardial Infarction: A Pilot Study. Prog Rehabil Med (2021) 6:20210017. doi: 10.2490/prm.20210017 33768186 PMC7972949

[50] Seok H. A New Member of Myocardial Ischemia-Reperfusion (MI/R) Associated miRNAs, miR-484: Its Potential Cardiac Protection Role. Korean Circ J (2020) 50(3):264–6. doi: 10.4070/kcj.2019.0413 PMC7043955 32100482

[51] Wang XC Tian LL Fan CX Duo CH Xu KM. The Adaptive Responses in Non-Small Cell Lung Cancer A549 Cell Lines Induced by Low-Dose Ionizing Radiation and the Variations of miRNA Expression. Dose Response (2021) 19(4):1485789883. doi: 10.1177/15593258211039931 PMC8516394 34658683

[52] Abend M Azizova T Muller K Dorr H Doucha-Senf S Kreppel H. Association of Radiation-Induced Genes With Noncancer Chronic Diseases in Mayak Workers Occupationally Exposed to Prolonged Radiation. Radiat Res (2015) 183(3):249–61. doi: 10.1667/RR13758.1 25706777

[53] Wang Y Zhu J Xu J Du J Lu X. The Long non-Coding RNA and mRNA Expression Profiles in Keratinocytes From Patients With Psoriasis Vulgaris. Ann Palliat Med (2021) 10(8):9206–14. doi: 10.21037/apm-21-2046 34488406

[54] Sheybani N Bakhtiarizadeh MR Salehi A. An Integrated Analysis of mRNAs, lncRNAs, and miRNAs Based on Weighted Gene Co-Expression Network Analysis Involved in Bovine Endometritis. Sci Rep (2021) 11(1):18050. doi: 10.1038/s41598-021-97319-y 34508138 PMC8433134

[55] Wu W Xiao H Laguna-Fernandez A Villarreal GJ Wang KC Geary GG. Flow-Dependent Regulation of Kruppel-Like Factor 2 Is Mediated by MicroRNA-92a. Circulation (2011) 124(5):633–41. doi: 10.1161/CIRCULATIONAHA.110.005108 PMC3511909 21768538

[56] Wang HW Lo HH Chiu YL Chang SJ Huang PH Liao KH. Dysregulated miR-361-5p/VEGF Axis in the Plasma and Endothelial Progenitor Cells of Patients With Coronary Artery Disease. PloS One (2014) 9(5):e98070. doi: 10.1371/journal.pone.0098070 24865854 PMC4035317

[57] Tang X Muniappan L Tang G Ozcan S. Identification of Glucose-Regulated miRNAs From Pancreatic {Beta} Cells Reveals a Role for miR-30d in Insulin Transcription. Rna (2009) 15(2):287–93. doi: 10.1261/rna.1211209 PMC2648717 19096044

[58] Raitoharju E Seppala I Oksala N Lyytikainen LP Raitakari O Viikari J. Blood microRNA Profile Associates With the Levels of Serum Lipids and Metabolites Associated With Glucose Metabolism and Insulin Resistance and Pinpoints Pathways Underlying Metabolic Syndrome: The Cardiovascular Risk in Young Finns Study. Mol Cell Endocrinol (2014) 391(1-2):41–9. doi: 10.1016/j.mce.2014.04.013 24784704

[59] Marzano F Faienza MF Caratozzolo MF Brunetti G Chiara M Horner DS. Pilot Study on Circulating miRNA Signature in Children With Obesity Born Small for Gestational Age and Appropriate for Gestational Age. Pediatr Obes (2018) 13(12):803–11. doi: 10.1111/ijpo.12439 30160046

[60] Choi WH Ahn J Um MY Jung CH Jung SE Ha TY. Circulating microRNA Expression Profiling in Young Obese Korean Women. Nutr Res Pract (2020) 14(4):412–22. doi: 10.4162/nrp.2020.14.4.412 PMC7390734 32765820

[61] Miyamoto Y Mauer AS Kumar S Mott JL Malhi H. Mmu-miR-615-3p Regulates Lipoapoptosis by Inhibiting C/EBP Homologous Protein. PloS One (2014) 9(10):e109637. doi: 10.1371/journal.pone.0109637 25314137 PMC4196923

[62] Rani N Nowakowski TJ Zhou H Godshalk SE Lisi V Kriegstein AR. A Primate lncRNA Mediates Notch Signaling During Neuronal Development by Sequestering miRNA. Neuron (2016) 90(6):1174–88. doi: 10.1016/j.neuron.2016.05.005 PMC4911262 27263970

[63] Leidinger P Backes C Deutscher S Schmitt K Mueller SC Frese K. A Blood Based 12-miRNA Signature of Alzheimer Disease Patients. Genome Biol (2013) 14(7):R78. doi: 10.1186/gb-2013-14-7-r78 23895045 PMC4053778

[64] Lee JH Choi JH Chueng SD Pongkulapa T Yang L Cho HY. Nondestructive Characterization of Stem Cell Neurogenesis by a Magneto-Plasmonic Nanomaterial-Based Exosomal miRNA Detection. ACS Nano (2019) 13(8):8793–803. doi: 10.1021/acsnano.9b01875 31361458

[65] Luceri C Bigagli E Pitozzi V Giovannelli L. A Nutrigenomics Approach for the Study of Anti-Aging Interventions: Olive Oil Phenols and the Modulation of Gene and microRNA Expression Profiles in Mouse Brain. Eur J Nutr (2017) 56(2):865–77. doi: 10.1007/s00394-015-1134-4 26695409

[66] Solich J Kolasa M Faron-Gorecka A Hajto J Piechota M Dziedzicka-Wasylewska M. MicroRNA Let-7e in the Mouse Prefrontal Cortex Differentiates Restraint-Stress-Resilient Genotypes From Susceptible Genotype. Int J Mol Sci (2021) 22(17):9439. doi: 10.3390/ijms22179439 34502349 PMC8430919

[67] Wingo TS Yang J Fan W Min CS Gerasimov ES Lori A. Brain microRNAs Associated With Late-Life Depressive Symptoms Are Also Associated With Cognitive Trajectory and Dementia. NPJ Genom Med (2020) 5:6. doi: 10.1038/s41525-019-0113-8 32047652 PMC7004995

[68] Cai Y Sun Z Jia H Luo H Ye X Wu Q. Rpph1 Upregulates CDC42 Expression and Promotes Hippocampal Neuron Dendritic Spine Formation by Competing With miR-330-5p. Front Mol Neurosci (2017) 10:27. doi: 10.3389/fnmol.2017.00027 28223918 PMC5293807

[69] Devaux Y Badimon L. CDR132L: Another Brick in the Wall Towards the Use of miRNAs to Treat Cardiovascular Disease. Eur Heart J (2021) 42(2):202–4. doi: 10.1093/eurheartj/ehaa870 33147612

[70] Sucharov C Bristow MR Port JD. miRNA Expression in the Failing Human Heart: Functional Correlates. J Mol Cell Cardiol (2008) 45(2):185–92. doi: 10.1016/j.yjmcc.2008.04.014 PMC2561965 18582896

[71] Su HH Liao JM Wang YH Chen KM Lin CW Lee IH. Exogenous GDF11 Attenuates Non-Canonical TGF-Beta Signaling to Protect the Heart From Acute Myocardial Ischemia-Reperfusion Injury. Basic Res Cardiol (2019) 114(3):20. doi: 10.1007/s00395-019-0728-z 30900023

[72] Wu HY Wu JL Ni ZL. Overexpression of microRNA-202-3p Protects Against Myocardial Ischemia-Reperfusion Injury Through Activation of TGF-Beta1/Smads Signaling Pathway by Targeting TRPM6. Cell Cycle (2019) 18(5):621–37. doi: 10.1080/15384101.2019.1580494 PMC6464590 30810438

[73] Caparosa EM Sedgewick AJ Zenonos G Zhao Y Carlisle DL Stefaneanu L. Regional Molecular Signature of the Symptomatic Atherosclerotic Carotid Plaque. Neurosurgery (2019) 85(2):E284–93. doi: 10.1093/neuros/nyy470 PMC12311974 30335165

[74] Li J Li L Li X Wu S. Long Noncoding RNA LINC00339 Aggravates Doxorubicin-Induced Cardiomyocyte Apoptosis by Targeting MiR-484. Biochem Biophys Res Commun (2018) 503(4):3038–43. doi: 10.1016/j.bbrc.2018.08.090 30170730

[75] Singh AK Rooge SB Varshney A Vasudevan M Kumar M Geffers R. Identification of miRNAs Associated With Dendritic Cell Dysfunction During Acute and Chronic Hepatitis B Virus Infection. J Med Virol (2021) 93(6):3697–706. doi: 10.1002/jmv.26629 33107616

[76] El-Maraghy SA Adel O Zayed N Yosry A El-Nahaas SM Gibriel AA. Circulatory miRNA-484, 524, 615 and 628 Expression Profiling in HCV Mediated HCC Among Egyptian Patients; Implications for Diagnosis and Staging of Hepatic Cirrhosis and Fibrosis. J Adv Res (2020) 22:57–66. doi: 10.1016/j.jare.2019.12.002 31956442 PMC6961223

[77] Castrillon-Betancur JC Urcuqui-Inchima S. Overexpression of miR-484 and miR-744 in Vero Cells Alters Dengue Virus Replication. Mem Inst Oswaldo Cruz (2017) 112(4):281–91. doi: 10.1590/0074-02760160404 PMC5354610 28327787

[78] Alipoor SD Mortaz E Tabarsi P Marjani M Varahram M Folkerts G. miR-1224 Expression Is Increased in Human Macrophages After Infection With Bacillus Calmette-Guerin (BCG). Iran J Allergy Asthma Immunol (2018) 17(3):250–7. 29908542

[79] Soares CT Trombone A Fachin L Rosa PS Ghidella CC Ramalho RF. Differential Expression of MicroRNAs in Leprosy Skin Lesions. Front Immunol (2017) 8:1035. doi: 10.3389/fimmu.2017.01035 28970833 PMC5609578

[80] Santulli G. microRNAs Distinctively Regulate Vascular Smooth Muscle and Endothelial Cells: Functional Implications in Angiogenesis, Atherosclerosis, and In-Stent Restenosis. Adv Exp Med Biol (2015) 887:53–77. doi: 10.1007/978-3-319-22380-3_4 26662986 PMC4871245

[81] Thounaojam MC Jadeja RN Warren M Powell FL Raju R Gutsaeva D. MicroRNA-34a (miR-34a) Mediates Retinal Endothelial Cell Premature Senescence Through Mitochondrial Dysfunction and Loss of Antioxidant Activities. Antioxidants (Basel) (2019) 8(9):328. doi: 10.3390/antiox8090328 PMC6769710 31443378

[82] Yang S Mi X Chen Y Feng C Hou Z Hui R. MicroRNA-216a Induces Endothelial Senescence and Inflammation *via* Smad3/IkappaBalpha Pathway. J Cell Mol Med (2018) 22(5):2739–49. doi: 10.1111/jcmm.13567 PMC5908109 29512862

[83] Raftrey B Williams I Rios CP Fan X Chang AH Zhao M. Dach1 Extends Artery Networks and Protects Against Cardiac Injury. Circ Res (2021) 129(7):702–16. doi: 10.1161/CIRCRESAHA.120.318271 PMC8448957 34383559

[84] Chang AH Raftrey BC D’Amato G Surya VN Poduri A Chen HI. DACH1 Stimulates Shear Stress-Guided Endothelial Cell Migration and Coronary Artery Growth Through the CXCL12-CXCR4 Signaling Axis. Genes Dev (2017) 31(13):1308–24. doi: 10.1101/gad.301549.117 PMC5580653 28779009

[85] MacGrogan D de la Pompa JL. DACH1-Driven Arterialization: Angiogenic Therapy for Ischemic Heart Disease? Circ Res (2021) 129(7):717–9. doi: 10.1161/CIRCRESAHA.121.319982 34529452

[86] Telkoparan-Akillilar P Cevik D. Identification of miR-17, miR-21, miR-27a, miR-106b and miR-222 as Endoplasmic Reticulum Stress-Related Potential Biomarkers in Circulation of Patients With Atherosclerosis. Mol Biol Rep (2021) 48(4):3503–13. doi: 10.1007/s11033-021-06352-7 33860430

[87] Larche J Lancel S Hassoun SM Favory R Decoster B Marchetti P. Inhibition of Mitochondrial Permeability Transition Prevents Sepsis-Induced Myocardial Dysfunction and Mortality. J Am Coll Cardiol (2006) 48(2):377–85. doi: 10.1016/j.jacc.2006.02.069 16843190

[88] Hollenberg SM Singer M. Pathophysiology of Sepsis-Induced Cardiomyopathy. Nat Rev Cardiol (2021) 18(6):424–34. doi: 10.1038/s41569-020-00492-2 33473203

[89] Hu X Miao H. MiR-539-5p Inhibits the Inflammatory Injury in Septic H9c2 Cells by Regulating IRAK3. Mol Biol Rep (2022) 49(1):121–30. doi: 10.1007/s11033-021-06849-1 PMC8748371 34757596

[90] Wang X Huang W Yang Y Wang Y Peng T Chang J. Loss of duplexmiR-223 (5p and 3p) Aggravates Myocardial Depression and Mortality in Polymicrobial Sepsis. Biochim Biophys Acta (2014) 1842(5):701–11. doi: 10.1016/j.bbadis.2014.01.012 PMC3959587 24486439

[91] Wang D Zhang Y Xu X Wu J Peng Y Li J. YAP Promotes the Activation of NLRP3 Inflammasome *via* Blocking K27-Linked Polyubiquitination of NLRP3. Nat Commun (2021) 12(1):2674. doi: 10.1038/s41467-021-22987-3 33976226 PMC8113592

[92] Lv Y Kim K Sheng Y Cho J Qian Z Zhao YY. YAP Controls Endothelial Activation and Vascular Inflammation Through Traf6. Circ Res (2018) 123(1):43–56. doi: 10.1161/CIRCRESAHA.118.313143 29794022 PMC6014930

[93] Lenneman CG Sawyer DB. Cardio-Oncology: An Update on Cardiotoxicity of Cancer-Related Treatment. Circ Res (2016) 118(6):1008–20. doi: 10.1161/CIRCRESAHA.115.303633 26987914

[94] Vejpongsa P Yeh ET. Prevention of Anthracycline-Induced Cardiotoxicity: Challenges and Opportunities. J Am Coll Cardiol (2014) 64(9):938–45. doi: 10.1016/j.jacc.2014.06.1167 25169180

[95] Bradshaw NJ Ukkola-Vuoti L Pankakoski M Zheutlin AB Ortega-Alonso A Torniainen-Holm M. The NDE1 Genomic Locus Can Affect Treatment of Psychiatric Illness Through Gene Expression Changes Related to microRNA-484. Open Biol (2017) 7(11):170153. doi: 10.1098/rsob.170153 29142105 PMC5717342

[96] Ceylan D Tufekci KU Keskinoglu P Genc S Ozerdem A. Circulating Exosomal microRNAs in Bipolar Disorder. J Affect Disord (2020) 262:99–107. doi: 10.1016/j.jad.2019.10.038 31726266

[97] Kumar M Bansal N. Implications of Phosphoinositide 3-Kinase-Akt (PI3K-Akt) Pathway in the Pathogenesis of Alzheimer’s Disease. Mol Neurobiol (2022) 59(1):354–85. doi: 10.1007/s12035-021-02611-7 34699027

[98] Li X Han Y Li D Yuan H Huang S Chen X. Identification and Validation of a Dysregulated miRNA-Associated mRNA Network in Temporal Lobe Epilepsy. BioMed Res Int (2021) 2021:4118216. doi: 10.1155/2021/4118216 34722763 PMC8556104

[99] Wu JS Cheung WM Tsai YS Chen YT Fong WH Tsai HD. Ligand-Activated Peroxisome Proliferator-Activated Receptor-Gamma Protects Against Ischemic Cerebral Infarction and Neuronal Apoptosis by 14-3-3 Epsilon Upregulation. Circulation (2009) 119(8):1124–34. doi: 10.1161/CIRCULATIONAHA.108.812537 PMC4144045 19221220

[100] Sajja V Jablonska A Haughey N Bulte J Stevens RD Long JB. Sphingolipids and microRNA Changes in Blood Following Blast Traumatic Brain Injury: An Exploratory Study. J Neurotrauma (2018) 35(2):353–61. doi: 10.1089/neu.2017.5009 29020847

[101] Sonkoly E Stahle M Pivarcsi A. MicroRNAs and Immunity: Novel Players in the Regulation of Normal Immune Function and Inflammation. Semin Cancer Biol (2008) 18(2):131–40. doi: 10.1016/j.semcancer.2008.01.005 18291670

[102] Fischer T Spohn M Olearo F Zinser ME Kasonta R Stubbe HC. Dynamic Changes of Circulating miRNAs Induced by the Ebola Virus Vaccine VSV-EBOV. Vaccine (2018) 36(46):7083–94. doi: 10.1016/j.vaccine.2018.09.016 30244872

[103] Alipoor SD Tabarsi P Varahram M Movassaghi M Dizaji MK Folkerts G. Serum Exosomal miRNAs Are Associated With Active Pulmonary Tuberculosis. Dis Markers (2019) 2019:1907426. doi: 10.1155/2019/1907426 30886653 PMC6388314

[104] Li Y Liu Y Yao J Li R Fan X. Downregulation of miR-484 Is Associated With Poor Prognosis and Tumor Progression of Gastric Cancer. Diagn Pathol (2020) 15(1):25. doi: 10.1186/s13000-020-00946-8 32192507 PMC7082931

[105] Lu X Lu J. The Significance of Detection of Serum miR-423-5p and miR-484 for Diagnosis of Colorectal Cancer. Clin Lab (2015) 61(1-2):187–90. doi: 10.7754/clin.lab.2014.140625 25807655

[106] Ma J Sun S Song C Li N Li N Xu L. Screening Potential microRNAs Associated With Pancreatic Cancer: Data Mining Based on RNA Sequencing and Microarrays. Exp Ther Med (2020) 20(3):2705–15. doi: 10.3892/etm.2020.8991 PMC7401655 32765765

[107] Li A Yu J Kim H Wolfgang CL Canto MI Hruban RH. MicroRNA Array Analysis Finds Elevated Serum miR-1290 Accurately Distinguishes Patients With Low-Stage Pancreatic Cancer From Healthy and Disease Controls. Clin Cancer Res (2013) 19(13):3600–10. doi: 10.1158/1078-0432.CCR-12-3092 PMC3707520 23697990

[108] Guo X Han T Hu P Guo X Zhu C Wang Y. Five microRNAs in Serum as Potential Biomarkers for Prostate Cancer Risk Assessment and Therapeutic Intervention. Int Urol Nephrol (2018) 50(12):2193–200. doi: 10.1007/s11255-018-2009-4 PMC6267169 30324582

[109] Haj-Ahmad TA Abdalla MA Haj-Ahmad Y. Potential Urinary miRNA Biomarker Candidates for the Accurate Detection of Prostate Cancer Among Benign Prostatic Hyperplasia Patients. J Cancer (2014) 5(3):182–91. doi: 10.7150/jca.6799 PMC3931266 24563673

[110] Merhautova J Hezova R Poprach A Kovarikova A Radova L Svoboda M. miR-155 and miR-484 Are Associated With Time to Progression in Metastatic Renal Cell Carcinoma Treated With Sunitinib. BioMed Res Int (2015) 2015:941980. doi: 10.1155/2015/941980 26064968 PMC4433647

[111] Prior C Perez-Gracia JL Garcia-Donas J Rodriguez-Antona C Guruceaga E Esteban E. Identification of Tissue microRNAs Predictive of Sunitinib Activity in Patients With Metastatic Renal Cell Carcinoma. PloS One (2014) 9(1):e86263. doi: 10.1371/journal.pone.0086263 24475095 PMC3901669

[112] Zhuang Z Sun C Gong H. High Serum miR-484 Expression Is Associated With the Diagnosis and Prognosis of Patients With Non-Small Cell Lung Cancer. Exp Ther Med (2019) 18(5):4095–102. doi: 10.3892/etm.2019.8010 PMC6796352 31641384

[113] Xue X Wang C Xue Z Wen J Han J Ma X. Exosomal miRNA Profiling Before and After Surgery Revealed Potential Diagnostic and Prognostic Markers for Lung Adenocarcinoma. Acta Biochim Biophys Sin (Shanghai) (2020) 52(3):281–93. doi: 10.1093/abbs/gmz164 32073597

[114] Ak G Tomaszek SC Kosari F Metintas M Jett JR Metintas S. MicroRNA and mRNA Features of Malignant Pleural Mesothelioma and Benign Asbestos-Related Pleural Effusion. BioMed Res Int (2015) 2015:635748. doi: 10.1155/2015/635748 25756049 PMC4331157

[115] Laco J Kovarikova H Chmelarova M Vosmikova H Sieglova K Bubancova I. Analysis of DNA Methylation and microRNA Expression in NUT (Nuclear Protein in Testis) Midline Carcinoma of the Sinonasal Tract: A Clinicopathological, Immunohistochemical and Molecular Genetic Study. Neoplasma (2018) 65(1):113–23. doi: 10.4149/neo_2018_161122N581 29322795

[116] Ye FG Song CG Cao ZG Xia C Chen DN Chen L. Cytidine Deaminase Axis Modulated by miR-484 Differentially Regulates Cell Proliferation and Chemoresistance in Breast Cancer. Cancer Res (2015) 75(7):1504–15. doi: 10.1158/0008-5472.CAN-14-2341 25643696

[117] Ning R Meng S Wang L Jia Y Tang F Sun H. 6 Circulating miRNAs can be Used as Non-Invasive Biomarkers for the Detection of Cervical Lesions. J Cancer (2021) 12(17):5106–13. doi: 10.7150/jca.51141 PMC8317520 34335927

[118] Oliveira D Carlsen AL Heegaard N Prahm KP Christensen IJ Hogdall CK. Diagnostic Plasma miRNA-Profiles for Ovarian Cancer in Patients With Pelvic Mass. PloS One (2019) 14(11):e225249. doi: 10.1371/journal.pone.0225249 PMC6860451 31738788

[119] Zhang W Su X Li S Liu Z Wang Q Zeng H. Low Serum Exosomal miR-484 Expression Predicts Unfavorable Prognosis in Ovarian Cancer. Cancer Biomark (2020) 27(4):485–91. doi: 10.3233/CBM-191123 PMC12662311 32065786

[120] Stokowy T Wojtas B Jarzab B Krohn K Fredman D Dralle H. Two-miRNA Classifiers Differentiate Mutation-Negative Follicular Thyroid Carcinomas and Follicular Thyroid Adenomas in Fine Needle Aspirations With High Specificity. Endocrine (2016) 54(2):440–7. doi: 10.1007/s12020-016-1021-7 27473101

[121] Luo H Ye Z. Identification of Serum miR-337-3p, miR-484, miR-582, and miR-3677 as Promising Biomarkers for Osteosarcoma. Clin Lab (2021) 67(4). doi: 10.7754/Clin.Lab.2020.200455 33865271

[122] Chai J Wang S Han D Dong W Xie C Guo H. MicroRNA-455 Inhibits Proliferation and Invasion of Colorectal Cancer by Targeting RAF Proto-Oncogene Serine/Threonine-Protein Kinase. Tumour Biol (2015) 36(2):1313–21. doi: 10.1007/s13277-014-2766-3 25355599

[123] Qi X Lin Y Liu X Chen J Shen B. Biomarker Discovery for the Carcinogenic Heterogeneity Between Colon and Rectal Cancers Based on lncRNA-Associated ceRNA Network Analysis. Front Oncol (2020) 10:535985. doi: 10.3389/fonc.2020.535985 33194594 PMC7662689

[124] Tessitore A Cicciarelli G Del VF Gaggiano A Verzella D Fischietti M. MicroRNA Expression Analysis in High Fat Diet-Induced NAFLD-NASH-HCC Progression: Study on C57BL/6J Mice. BMC Cancer (2016) 16:3. doi: 10.1186/s12885-015-2007-1 26728044 PMC4700747

[125] Jia YZ Miao QJ Zhang Y. miR-484 Mediates Apoptotic Cell Death by Targeting SIRT1 in Nonalcoholic Fatty Liver Disease Injury. World J Chin Digestion 389-97 (2021) 29(08):389–97. (in Chinese). doi: 10.11569/wcjd.v29.i8.389

[126] Pashaei E Pashaei E Ahmady M Ozen M Aydin N. Meta-Analysis of miRNA Expression Profiles for Prostate Cancer Recurrence Following Radical Prostatectomy. PloS One (2017) 12(6):e179543. doi: 10.1371/journal.pone.0179543 PMC5484492 28651018

[127] Yao Z Zhang Q Guo F Guo S Yang B Liu B. Long Noncoding RNA PCED1B-AS1 Promotes the Warburg Effect and Tumorigenesis by Upregulating HIF-1alpha in Glioblastoma. Cell Transplant (2020) 29:2138899481. doi: 10.1177/0963689720906777 PMC7444212 32326742

[128] Kovacova J Juracek J Poprach A Buchler T Kopecky J Fiala O. Candidate MicroRNA Biomarkers of Therapeutic Response to Sunitinib in Metastatic Renal Cell Carcinoma: A Validation Study in Patients With Extremely Good and Poor Response. Anticancer Res (2018) 38(5):2961–5. doi: 10.21873/anticanres.12546 29715124

[129] Shi W Dong F Jiang Y Lu L Wang C Tan J. Construction of Prognostic microRNA Signature for Human Invasive Breast Cancer by Integrated Analysis. Onco Targets Ther (2019) 12:1979–2010. doi: 10.2147/OTT.S189265 30936717 PMC6430069

[130] Volinia S Croce CM. Prognostic microRNA/mRNA Signature From the Integrated Analysis of Patients with Invasive Breast Cancer. Proc Natl Acad Sci (2013) 110(18):7413–7. doi: 10.1073/pnas.1304977110 PMC3645522 23589849

[131] Zearo S Kim E Zhu Y Zhao JT Sidhu SB Robinson BG. MicroRNA-484 Is More Highly Expressed in Serum of Early Breast Cancer Patients Compared to Healthy Volunteers. BMC Cancer (2014) 14:200. doi: 10.1186/1471-2407-14-200 24641801 PMC3995145

[132] Holubekova V Kolkova Z Grendar M Brany D Dvorska D Stastny I. Pathway Analysis of Selected Circulating miRNAs in Plasma of Breast Cancer Patients: A Preliminary Study. Int J Mol Sci (2020) 21(19):7288. doi: 10.3390/ijms21197288 PMC7583045 33023154

[133] Ritter A Hirschfeld M Berner K Jaeger M Grundner-Culemann F Schlosser P. Discovery of Potential Serum and Urine-Based microRNA as Minimally-Invasive Biomarkers for Breast and Gynecological Cancer. Cancer biomark (2020) 27(2):225–42. doi: 10.3233/CBM-190575 PMC12662288 32083575

[134] Xie JX Fan X Drummond CA Majumder R Xie Y Chen T. MicroRNA Profiling in Kidney Disease: Plasma Versus Plasma-Derived Exosomes. Gene (2017) 627:1–8. doi: 10.1016/j.gene.2017.06.003 28587849 PMC5534180

