# Supplementary material for: miR-484: A Potential Biomarker in Health and Disease
Source: Front Oncol. 2022 Mar 9;12:830420. doi: 10.3389/fonc.2022.830420 (PMC8959652; doi:10.3389/fonc.2022.830420)
Supplement: Supplementary file 1 [file Table_1.docx]

Supplementary Material

# Supplementary Tables 1 The predicted target of has-miR-484

| **Database** | **Target Genes Number** | **Target Genes Name** | | | | | | | |
| --- | --- | --- | --- | --- | --- | --- | --- | --- | --- |
| DIANA-microT  miRanda  PITA  TargetScan | 49 | | N4BP2L2  ZNF25  SCP2  ZNF667  LAMB3  HOXA5  BSDC1 | POLD4  TK2  CREM  AVL9  WFS1  GRAMD1C  MTF2 | TCHP  PDGFA  TARBP2  ZNF688  SPSB4  LPL  SNRNP200 | NCAN  FLVCR2  DPYSL2  CCR9  RBM24  MED8  TRAT1 | LTBP2  MAP10  ANO2  SMIM7  STARD3NL  WASF3  ARFIP2 | MAP4K5  CLEC6A  ADAM33  HIVEP2  SOX5  FGF7  SLC20A2 | ACVR1B  IL20RB  TNFSF9  EIF4G2  CSRNP2  CLDN18 |
| miRanda  PITA  TargetScan | 15 | | BIRC5  UPF3A  FAM120A | GAP43  DCAF4 | RFC1  PER1 | SENP1  GRIPAP1 | SLC52A3  ACHE | TBL1X  GTDC1 | DGCR6  UNC13C |
| DIANA-microT  PITA  TargetScan | 134 | | SEMA4D  MAX  SUSD2  TCEANC2  CACTIN  OGDH  CPLX3  ABR  LAIR1  FBXO31  CLCN4  TACR1  CNTFR  LGI4  IL6R  HPS4  HNRNPUL2  SNN  DOK4  SLC17A9 | LIN28B  VTI1A  LDLR  LMAN2L  XPO4  IFNAR1  C1QTNF7  IL21R  NT5E  HERC3  KRT74  RASGEF1A  STX5  CSRNP1  PIK3CD  RUNDC1  FILIP1  CRTAP  C3orf62 | PPARD  CARHSP1  SH3TC2  GJD4  DDX31  KLHDC3  CYB5B  DNAJB7  METTL6  FCF1  TGFBRAP1  EIF3J  APLN  ARSD  SH2D1B  BCL7B  GJC1  STAM2  CHURC1 | RNF8  ST6GAL1  TSTD2  AMPD2  CALM1  MAP3K11  RFC5  COLQ  TMEM130  ALPK3  LYRM2  MAB21L3  NGRN  UAP1L1  ZNF581  DCBLD2  GPR173  STEAP3  SEC31B | TRMT10B  GDI1  KHK  CCDC120  VAPB  MRFAP1L1  MAGIX  PHF19  TNK2  CRTC2  TAF1L  HHIPL2  PNRC1  PYGO2  NDOR1  TINF2  ABLIM2  SERPINF2  PICK1 | EXOSC5  EMX1  CCDC142  ANGPT2  RIN1  BRD9  FOSL2  GRB10  MEN1  NUBPL  C10orf91  CDH16  SORBS1  PEA15  TOX4  FNDC5  LAMTOR1  HK2  IGLON5 | GTPBP10  PAIP2B  KLHDC10  CBLN3  CYB5RL  SLC41A3  CRTC3  CYB561D1  LYSMD1  YAP1  CPSF4  WDR86  F2RL3  CABLES1  CTAGE1  FAM110B  C3orf38  FXYD3  WASF2 |
| DIANA-microT  miRanda  TargetScan | 44 | | DKK2  VKORC1  SEC23IP  FAM71E2  SORBS2  CCDC77  XKR9 | C6orf141  PRM1  VCP  PTGER4  POT1  GCSAM  SLC6A1 | DNAJC5G  TOMM5  EME1  IGBP1  RABGAP1L  HLA-DOB | HOXA11  C10orf113  RHOBTB1  MBD1  TOPBP1  PTPRE | RTN3  ANKAR  EPHA5  TSGA10  THBD  ANAPC7 | NBPF20  NCR3  TPRXL  KCNJ14  TPRX1  CDK9 | CCNL1  MYCBP2  NUP54  FLOT1  ARL15  NBPF15 |
| DIANA-microT  miRanda  PITA | 19 | | FAM134B  PIKFYVE  KIAA1033 | NPNT  KDM4A  XPNPEP3 | FCRL5  GOSR2  GPR63 | SAMD4B  CSF1  REV3L | CCDC53  KIAA1549  RANBP17 | TRIM71  MAP2 | KIAA0430  ZNF37A |

**Supplementary Table 2 The predicted target of mus-miR-484**

| **Database** | **Target Genes Number** | **Target Genes Name** | | | | | | |
| --- | --- | --- | --- | --- | --- | --- | --- | --- |
| DIANA-microT  miRanda  PITA  TargetScan | 45 | Snrk  Tsx  Efna1  Serpina3n  Wdr78  Tm4sf4  Snn | Fam76b  Dcaf8  Cyb5d2  Hook2  Ccr5  Mcfd2  Tcl1b2 | Ggta1  Med8  Gk5  Slc6a1  Gabpa  Mmab  Plod2 | Map3k5  Csf1  Ints2  Sorbs2  Zwilch  Prox1 | Stt3b  Hip1r  Ptpre  Hnf1a  Add2  4932411E22Rik | Slc12a4  Ranbp6  Slc4a4  Sdhaf1  Hoxa5  Susd2 | Olfml1  Lhfp  Dpysl2  Hivep2  Ltbp2  Ppp1r3d |
| miRanda  PITA  TargetScan | 28 | Dhx9  Tor3a  Magi1  Gcdh | Eif3j1  Arpc3  Atxn7  Fam120a | Slc7a6  Zic5  Pctp  Dmbt1 | Rnf8  Slit2  Mzt1  Anln | Itsn1  St6galnac2  Pik3r6  Entpd4 | Fam110c  Gtdc1  Abcb10  Ppil1 | Trhr  Arf6  Chchd3  Pecam1 |
| DIANA-microT  PITA  TargetScan | 136 | Parp1  Nkain1  Cd274  Rnf24  Cacfd1  Mpp2  Tspan17  Gnaq  Bcl2l1  Scarb1  Thbd  Map2  Igfbp4  Eif4g2  Htt  Stard13  Tcl1b1  Lrrc20  Ehbp1l1  Kctd11 | Ikzf1  Tbc1d14  Zcchc17  Ddit4l  Kcnq1  Tpk1  Agtrap  Hspa12b  Dnajc18  Csdc2  Kcng1  Phospho2  Lrrc2  Plk3  Panx2  Frmd8  Prrt2  Tmem44  Ska2  Ticrr | Pgbd5  Cln8  Cyp1b1  Sec24c  Pigq  Fbxl2  Lrrc3  Kctd14  Tbc1d13  Smarcc1  Stil  Serpinb9  Dhrs3  E430018J23Rik  Atp5g1  Sstr3  Srebf1  Fam13a  Tmem69  Acvrl1 | Fbxw8  Crtc2  7420426K07Rik  Gtpbp10  Dcp1a  Psme3  Acvr1b  Clock  Aar2  Abl1  Rab36  Mrpl11  Stk10  Slc16a3  Myo1d  Tcl1b3  Nfe2l1  Slco2b1  Nrbp2 | Sap130  Tmie  Limk2  Bdh1  Rhov  Rnase10  Ddx54  Sh2d2a  Zdhhc18  Mgrn1  Rcn1  Srrm4  Preb  Aoc3  Lgi4  D630039A03Rik  Skp2  Chrna6  Lasp1 | Herc2  Cntfr  Ythdf3  Il21r  Gm6878  Rgs9bp  8-Mar  Mob3c  Rnf14  Wnt8a  Scrn1  Med6  Mfap3  Gadd45a  Timp2  Pkp1  Sirt2  Cdc7  Stx5a | Tcl1b4  Ttc9  Pea15a  Elp4  Lamb3  Dlst  Elovl1  Grb10  Asb1  4930524B15Rik  Zfp667  Inpp4b  Rasal1  Bsdc1  Snx22  Emx1  Fam83a  Cep85  Lat |
| DIANA-microT  miRanda  TargetScan | 36 | Rbbp8nl  Mmrn1  Wfdc5  Trps1  Dll3  M6pr | Eogt  Pik3r5  Sh2b2  Asprv1  Mycbp2  Lrrc56 | Pds5b  Cnpy1  Trim52  Itga5  Zc3h6  Fbf1 | Spag11b  Rhpn2  Zswim6  Plekhf1  Emc6 | 0610030E20Rik  Gm4884  Nucks1  Ipo11  1700031F05Rik | Myb  Ibtk  Tgfbr3  Prm1  Dach1 | Mtf2  Ppl  Serpina3b  Rbm27  Maml2 |
| DIANA-microT  miRanda  PITA | 9 | Ppp4r1  Kcnk2 | Trim33  Apbb2 | Gm13102 | Strn | A230046K03Rik | Ccdc127 | Xpnpep3 |
